# Psychiatric and neuropsychiatric presentations associated with severe coronavirus infections: a systematic review and meta-analysis with comparison to the COVID-19 pandemic

**DOI:** 10.1016/S2215-0366(20)30203-0

**Published:** 2020-07

**Authors:** Jonathan P Rogers, Edward Chesney, Dominic Oliver, Thomas A Pollak, Philip McGuire, Paolo Fusar-Poli, Michael S Zandi, Glyn Lewis, Anthony S David

**Affiliations:** a Division of Psychiatry, University College London, London, UK; b South London and Maudsley NHS Foundation Trust, London, UK; c Department of Psychosis Studies, King's College London, London, UK; d Department of Brain and Behavioral Sciences, University of Pavia, Pavia, Italy; e UCL Queen Square Institute of Neurology, University College London, London, UK; f University College London Hospitals NHS Foundation Trust, London, UK; g UCL Institute of Mental Health, University College London, London, UK

## Abstract

**Background:**

Before the COVID-19 pandemic, coronaviruses caused two noteworthy outbreaks: severe acute respiratory syndrome (SARS), starting in 2002, and Middle East respiratory syndrome (MERS), starting in 2012. We aimed to assess the psychiatric and neuropsychiatric presentations of SARS, MERS, and COVID-19.

**Methods:**

In this systematic review and meta-analysis, MEDLINE, Embase, PsycINFO, and the Cumulative Index to Nursing and Allied Health Literature databases (from their inception until March 18, 2020), and medRxiv, bioRxiv, and PsyArXiv (between Jan 1, 2020, and April 10, 2020) were searched by two independent researchers for all English-language studies or preprints reporting data on the psychiatric and neuropsychiatric presentations of individuals with suspected or laboratory-confirmed coronavirus infection (SARS coronavirus, MERS coronavirus, or SARS coronavirus 2). We excluded studies limited to neurological complications without specified neuropsychiatric presentations and those investigating the indirect effects of coronavirus infections on the mental health of people who are not infected, such as those mediated through physical distancing measures such as self-isolation or quarantine. Outcomes were psychiatric signs or symptoms; symptom severity; diagnoses based on ICD-10, DSM-IV, or the Chinese Classification of Mental Disorders (third edition) or psychometric scales; quality of life; and employment. Both the systematic review and the meta-analysis stratified outcomes across illness stages (acute *vs* post-illness) for SARS and MERS. We used a random-effects model for the meta-analysis, and the meta-analytical effect size was prevalence for relevant outcomes, *I*^2^ statistics, and assessment of study quality.

**Findings:**

1963 studies and 87 preprints were identified by the systematic search, of which 65 peer-reviewed studies and seven preprints met inclusion criteria. The number of coronavirus cases of the included studies was 3559, ranging from 1 to 997, and the mean age of participants in studies ranged from 12·2 years (SD 4·1) to 68·0 years (single case report). Studies were from China, Hong Kong, South Korea, Canada, Saudi Arabia, France, Japan, Singapore, the UK, and the USA. Follow-up time for the post-illness studies varied between 60 days and 12 years. The systematic review revealed that during the acute illness, common symptoms among patients admitted to hospital for SARS or MERS included confusion (36 [27·9%; 95% CI 20·5–36·0] of 129 patients), depressed mood (42 [32·6%; 24·7–40·9] of 129), anxiety (46 [35·7%; 27·6–44·2] of 129), impaired memory (44 [34·1%; 26·2–42·5] of 129), and insomnia (54 [41·9%; 22·5–50·5] of 129). Steroid-induced mania and psychosis were reported in 13 (0·7%) of 1744 patients with SARS in the acute stage in one study. In the post-illness stage, depressed mood (35 [10·5%; 95% CI 7·5–14·1] of 332 patients), insomnia (34 [12·1%; 8·6–16·3] of 280), anxiety (21 [12·3%; 7·7–17·7] of 171), irritability (28 [12·8%; 8·7–17·6] of 218), memory impairment (44 [18·9%; 14·1–24·2] of 233), fatigue (61 [19·3%; 15·1–23·9] of 316), and in one study traumatic memories (55 [30·4%; 23·9–37·3] of 181) and sleep disorder (14 [100·0%; 88·0–100·0] of 14) were frequently reported. The meta-analysis indicated that in the post-illness stage the point prevalence of post-traumatic stress disorder was 32·2% (95% CI 23·7–42·0; 121 of 402 cases from four studies), that of depression was 14·9% (12·1–18·2; 77 of 517 cases from five studies), and that of anxiety disorders was 14·8% (11·1–19·4; 42 of 284 cases from three studies). 446 (76·9%; 95% CI 68·1–84·6) of 580 patients from six studies had returned to work at a mean follow-up time of 35·3 months (SD 40·1). When data for patients with COVID-19 were examined (including preprint data), there was evidence for delirium (confusion in 26 [65%] of 40 intensive care unit patients and agitation in 40 [69%] of 58 intensive care unit patients in one study, and altered consciousness in 17 [21%] of 82 patients who subsequently died in another study). At discharge, 15 (33%) of 45 patients with COVID-19 who were assessed had a dysexecutive syndrome in one study. At the time of writing, there were two reports of hypoxic encephalopathy and one report of encephalitis. 68 (94%) of the 72 studies were of either low or medium quality.

**Interpretation:**

If infection with SARS-CoV-2 follows a similar course to that with SARS-CoV or MERS-CoV, most patients should recover without experiencing mental illness. SARS-CoV-2 might cause delirium in a significant proportion of patients in the acute stage. Clinicians should be aware of the possibility of depression, anxiety, fatigue, post-traumatic stress disorder, and rarer neuropsychiatric syndromes in the longer term.

**Funding:**

Wellcome Trust, UK National Institute for Health Research (NIHR), UK Medical Research Council, NIHR Biomedical Research Centre at University College London Hospitals NHS Foundation Trust and University College London.

## Introduction

Viral infections are common and some are known to infect the CNS, causing neuropsychiatric syndromes affecting cognitive, affective, behavioural, and perceptual domains.^1^, ^2^, ^3^ Severe illness of diverse aetiologies is associated with subsequent psychiatric morbidity, at least some of which is attributable to its psychological impact of trauma.^4^, ^5^, ^6^

Coronaviruses are single-stranded RNA viruses and several subtypes affecting humans have been identified, most of which cause mild upper respiratory tract infections in immunocompetent individuals (notably, the HCoV-229E, HCoV-OC43, HCoV-NL63, and HCoV-HKU1 strains).^7^, ^8^ Coronaviruses have also been detected in both the brain and the cerebrospinal fluid of individuals with seizures, encephalitis, and encephalomyelitis.^9^ Novel strains of coronavirus caused the severe acute respiratory syndrome (SARS) outbreak, starting in 2002, and the Middle East respiratory syndrome (MERS) outbreak, starting in 2012.^8^

On Dec 31, 2019, WHO was made aware of several cases of atypical pneumonia in Wuhan, China, which were subsequently identified as being caused by a novel coronavirus termed severe acute respiratory syndrome coronavirus 2 (SARS-CoV-2;^10^ panel). As the pandemic of the disease now known as COVID-19 has spread, there has been a growing recognition of the psychiatric implications of the disease.^13^, ^14^ There are several reasons why the current COVID-19 pandemic might have psychiatric consequences. Some of these reasons relate to the wider social impact of the pandemic and the governmental response, including physical distancing measures and quarantine.^15^, ^16^ Both the infected and non-infected population might be susceptible as a result of certain experiences, such as widespread anxiety,^17^ social isolation,^16^ stress in health-care workers and other essential workers,^18^ and unemployment and financial difficulties.^19^ Other experiences might be specific to individuals who are infected with the virus, such as concern about the outcome of their illness,^20^ stigma,^21^ and amnesia or traumatic memories of severe illness.^22^

Panel **Terminology**

**Coronanavirus**

A group of viruses that predominantly cause mild upper respiratory tract infections in humans.

**Severe acute respiratory syndrome coronavirus (SARS-CoV)**

A clade I, cluster IIb betacoronavirus that enters host cells via the angiotensin-converting enzyme 2 receptor.

**Severe acute respiratory syndrome (SARS)**

The clinical syndrome associated with infection with SARS-CoV that emerged in humans in 2002, affecting approximately 8096 people.^11^

**Middle East respiratory syndrome coronavirus (MERS-CoV)**

A clade II betacoronavirus that enters host cells via the dipeptidyl peptidase 4 receptor.

**Middle East respiratory syndrome (MERS)**

The clinical syndrome associated with infection with MERS-CoV that emerged in humans in 2012, affecting approximately 2260 people.^11^

**Severe acute respiratory syndrome coronavirus 2 (SARS-CoV-2)**

A clade I, cluster IIa betacoronavirus with structural similarity to SARS-CoV that enters host cells via the angiotensin-converting enzyme 2 receptor.

**COVID-19**

The clinical syndrome—primarily a respiratory disorder—associated with infection with SARS-CoV-2.

**Psychiatric**

We use this term to include disorders, symptoms, and signs listed in category 06 (mental, behavioural, or neurodevelopmental disorders) of the 11th edition of the ICD.^12^

**Neuropsychiatric**

We use this term to denote psychiatric disorders, symptoms, and signs that are the result of brain damage or disease.

Neuropsychiatric consequences—ie, mental disorders that are the sequelae of brain damage or disease—can arise either through direct effects of infection of the CNS or indirectly via an immune response or medical therapy. A case series from Wuhan found that among patients admitted to hospital for infection with SARS-CoV-2, 36% had neurological features, mostly consisting of mild symptoms such as dizziness and headache, although these symptoms might be manifestations more of systemic illness than a specific neurological syndrome.^23^ Some patients had acute cerebrovascular disease or impaired consciousness as part of their illness.^23^ SARS-CoV-2 enters human host cells by the angiotensin-converting enzyme 2 receptor, which has little expression in the brain.^24^, ^25^ There has been speculation that other routes of CNS infiltration might account for the respiratory failure caused by infection with SARS-CoV-2, although there is currently no evidence.^24^ There is preliminary in-vitro evidence that—possibly unlike SARS coronavirus (SARS-CoV)—SARS-CoV-2 can replicate in neuronal cells, but the translation of this finding to in-vivo settings remains unclear.^26^ Even if severe neuropsychiatric consequences are proportionately rare, a considerable number of individuals worldwide would be affected.^27^, ^28^ Previous influenza pandemics have been associated with long-lasting neuropsychiatric consequences,^29^ so it is possible that other viral infections on a large scale could cause sustained mental morbidity.

**Research in context**

**Evidence before this study**

Severe acute respiratory syndrome (SARS) and Middle East respiratory syndrome (MERS) cause severe respiratory illness, and a few studies have examined the acute and post-illness psychiatric and neuropsychiatric outcomes of these diseases. The COVID-19 pandemic has affected a large proportion of the world's population, but relatively little is known about its potential direct effects on mental health. MEDLINE, Embase, PsycINFO, and the Cumulative Index to Nursing and Allied Health Literature databases were searched from inception to March 18, 2020, for terms relating to coronavirus infection and psychiatric presentations. medRxiv, bioRxiv, and PsyArXiv were searched for relevant preprints published between Jan 1, 2020, and April 10, 2020. Studies were included if they provided numerical or formal qualitative data on psychiatric presentations of coronavirus outbreaks. The majority of studies were of low or moderate quality.

**Added value of this study**

This systematic review and meta-analysis suggests that among patients admitted to hospital for severe SARS or MERS coronavirus infections, delirium is common acutely, whereas post-traumatic stress disorder, depression, anxiety, and fatigue are common in the following months. Preliminary data suggest patients with COVID-19 might experience delirium, confusion, agitation, and altered consciousness, as well as symptoms of depression, anxiety, and insomnia.

**Implications of all the available evidence**

Previous coronavirus epidemics were associated with a significant psychiatric burden in both the acute and post-illness stages. In the current COVID-19 pandemic, there is already evidence of delirium acutely and clinicians should be alert to the possibility of high rates of common mental disorders in the longer term. High-quality, peer-reviewed research into psychiatric symptoms of patients infected with SARS-CoV-2 as well as into potential mitigating factors and interventions is needed.

We are not aware of a systematic review of the psychiatric consequences of all forms of coronavirus infection, including the recent data on COVID-19, to inform clinicians of the possible longer-term consequences of this pandemic. We aimed to examine the two previous coronavirus epidemics, SARS and MERS, to identify the possible psychiatric and neuropsychiatric implications for the current pandemic. We also examined the early data from the COVID-19 outbreak.

## Methods

### Search strategy and selection criteria

In this systematic review and meta-analysis, we searched MEDLINE, Embase, PsycINFO, and the Cumulative Index to Nursing and Allied Health Literature databases for studies or abstracts published between database inception and March 18, 2020. We used a combined set of keywords (appendix pp 6–8) to identify human studies reporting on a broad range of psychiatric presentations, symptom severity, diagnoses, employment, and quality of life in association with coronavirus exposure. Neuropsychiatric concepts such as confusion and cognition were included, but we did not include neurological disorders such as stroke, seizure, and encephalomyelitis because these disorders do not necessarily have psychiatric presentations and any psychiatric presentations would be captured by the psychiatric search terms. Our definitions are included in the panel.

For MEDLINE, the terms were: (((coronavir* OR alphacoronavirus OR betacoronavirus OR COVID OR COVID-19 OR “severe acute respiratory syndrome” OR SARS OR “Middle East respiratory syndrome” OR MERS OR “infectious bronchitis vir*” OR “infectious bronchitis”).ti,ab OR (exp coronaviridae/ OR exp “severe acute respiratory syndrome”/)) AND ((deliri* OR sleep OR insomnia OR somnolence OR hypersomnolence OR parasomnia OR “movement disorder” OR neuropsych* OR dement* OR cogniti* OR irritability OR hallucinat* OR delusion* OR apath* OR indifference OR agitat* OR euphori* OR elation OR elated OR disinhibit* OR aggressi* OR amnes* OR catatoni* OR personality OR psycho* OR mental OR mood OR affective OR depress* OR anxi* OR “obsessive compulsive” OR OCD OR “panic disorder” OR post-trauma* OR posttrauma* OR PTSD OR neurosis OR neurotic OR bipolar OR mania OR manic OR schizophreni* OR “intelligence quotient” OR IQ OR “mental retardation” OR “intellectual disability” OR “learning disability” OR autis* OR asperger* OR “attention deficit” OR ADHD OR hyperactivity OR hyperkinetic OR suicid* OR emotion* OR appetite OR fatigu* OR tired* OR confus* OR “quality of life” OR QoL OR employment OR unemployment).ti,ab OR (exp delirium/ OR exp sleep/ OR exp wakefulness/ OR exp sleep/ OR exp “disorders of excessive somnolence”/ OR exp parasomnias/ OR exp “psychomotor disorders”/ OR exp dementia/ OR exp “neurocognitive disorders”/ OR exp hallucinations/ OR exp delusions/ OR exp apathy/ OR exp “psychomotor agitation”/ OR exp euphoria/ OR exp aggression/ OR exp amnesia/ OR exp catatonia/ OR exp “personality disorders”/ OR exp “schizophrenia spectrum and other psychotic disorders”/ OR exp “mental disorders”/ OR exp “mood disorders”/ OR exp depression/ OR exp anxiety/ OR exp “anxiety disorders”/ OR exp “obsessive-compulsive disorder”/ OR exp “panic disorder”/ OR exp “stress disorders, post-traumatic”/ OR exp “bipolar and related disorders”/ OR exp schizophrenia/ OR exp “intellectual disability”/ OR exp “autism spectrum disorder”/ OR exp “asperger syndrome”/ OR exp “attention deficit and disruptive behavior disorders”/ OR exp “attention deficit disorder with hyperactivity”/ OR exp “motor activity”/ OR exp suicide/ OR exp emotions/ OR exp appetite/ OR exp “FEEDING AND EATING DISORDERS”/ OR exp FATIGUE/ OR exp CONFUSION/ OR exp “quality of life”/ OR exp employment/ OR exp unemployment/))) [Humans].

Given that this field is developing rapidly, we also searched the preprint servers medRxiv, PsyArXiv, and bioRxiv for studies published between Jan 1, 2020, and April 10, 2020, with the terms “coronavirus” or “COVID-19” in the title or abstract. In addition, relevant experts in the field were individually contacted and the references of other review articles were examined.

Duplicate references were removed electronically and manually. Titles, abstracts, and full texts of articles were independently screened by two reviewers (JPR and EC). Where there was disagreement on the inclusion of a title or abstract, it was retained for the next stage of screening. Disagreement on the inclusion of a full-text article was discussed with an independent arbiter (DO). Reasons for exclusion of full texts were collected.

We included English-language studies that reported the psychiatric and neuropsychiatric features of suspected or confirmed cases of three types of coronavirus infection (SARS-CoV, MERS coronavirus [MERS-CoV], and SARS-CoV-2). Randomised controlled trials, cohort studies, case-control studies, cross-sectional studies, case series, case reports, and qualitative studies were included. Preprints and letters were included if they described original research that contained data on patients with suspected or laboratory-confirmed coronavirus infection, if data on individuals infected with coronavirus were distinguishable from data on any individuals not infected, and if specific neuropsychiatric features were listed, but conference abstracts were excluded because they lacked sufficient information for quality assessment and data extraction.

We excluded studies limited to neurological complications without specified neuropsychiatric presentations, but we included neuropsychiatric presentations (eg, cognitive impairment, apathy, insomnia, altered consciousness, and delirium). We excluded studies investigating the indirect effects of coronavirus infections on the mental health of people not infected mediated through physical distancing measures such as self-isolation or quarantine, because these have been recently appraised.^16^

This systematic review followed PRISMA guidelines (appendix pp 2–5), although the study protocol was not registered.

### Data extraction

Data were extracted by two of three independent reviewers (EC, DO, and JPR). Where relevant data were missing from a report, the author was contacted. Descriptive variables extracted were setting (ie, country), population type (eg, pregnant women and children), study design (eg, cohort and case-control), virus subtype (SARS-CoV, MERS-CoV, and SARS-CoV-2), diagnostic criteria for viral infection (eg, WHO guidelines), timing (acute *vs* post-illness), follow-up time, nature of the control group, number of cases, number of controls, age, and gender. Randomised controlled trials, for the purposes of this review, were treated as cohort studies. For example, if a trial investigated the effects of an antiviral medication versus placebo, data from all participants regardless of treatment group would be extracted together.

### Outcomes

Outcomes were divided into number of signs or symptoms; symptom severity (ie, anxiety, depression, or trauma); proportion of diagnoses (ie, anxiety, depression, and post-traumatic stress disorder); quality of life scores; and proportion of individuals employed. If more than one dataset was reported for the same group of patients, the outcomes that were assessed after the longest follow-up were used, and point prevalence values were used if available. Studies were categorised as examining the acute versus post-illness psychiatric consequences of infection on the basis of whether they collected information during the patient's illness or the period after the illness. Factors associated with the development of adverse outcomes were extracted and reported if odds ratios were reported or could be robustly calculated.

### Data analysis

The meta-analysis was planned for the proportion of psychiatric diagnoses; severity of anxiety, depression, and post-traumatic symptoms; quality of life; and proportion of individuals employed. We used a random-effects model because high heterogeneity was expected. The effect size measures were prevalence with 95% CIs (for number of signs or symptoms, quality of life scores, and employment) and mean difference with 95% CIs (for symptom severity and proportion of diagnoses). We defined point prevalences for number of psychiatric symptoms, proportion of diagnoses (defined by ICD-10, DSM-IV, or Chinese Classification of Mental Disorders [third edition] criteria or by validated psychometric scales with established cutoffs), and proportion of patients in employment as the proportion of cases over the sample size.^30^ For studies using cutoff scores on symptom rating scales, this percentage represents the presence of clinically significant symptoms reflected by the number of patients scoring above the defined cutoff. We also synthesised prevalences for individuals admitted to an intensive care unit (ICU) and undergoing mechanical ventilation for each coronavirus subtype for comparability. We used mean difference for symptom severity and quality-of-life outcomes, with negative values indexing lower symptom severity and higher quality of life, and positive values indexing higher symptom severity and lower quality of life, in patients with coronavirus infection than among healthy controls. Where continuous data (ie, symptom scores and quality of life) did not have a sufficient number of studies reporting suitable control group data to produce mean differences, we calculated sample size-weighted mean scores for all the studies reporting data alongside 95% CIs in addition to any potential meta-analytical summary effect. We calculated *I*^2^ as a measure of between-study heterogeneity. We did not assess funnel plot asymmetry because of an insufficient number of studies.^31^ Sensitivity analyses were done to assess the contribution of individual studies for the meta-analyses of diagnoses. Data were analysed using R (version 3.3.2) and the meta package (version 4.11) for prevalence data, and RevMan Web (version 5.3) for continuous outcomes. The threshold for significance was set to p values of less than 0·05.

To assess study quality, we adapted the Newcastle Ottawa Scale to enhance its relevance to the specific requirements of this review, such as including laboratory verification, as described in full in the appendix (pp 9–10).^32^

### Role of the funding source

The funders of the individuals working on the study had no role in study design, data collection, data analysis, data interpretation, or writing of the report. JPR, EC, and DO had access to the raw data. The corresponding author had full access to all the data in the study and had final responsibility for the decision to submit for publication.

## Results

**Figure 1:**
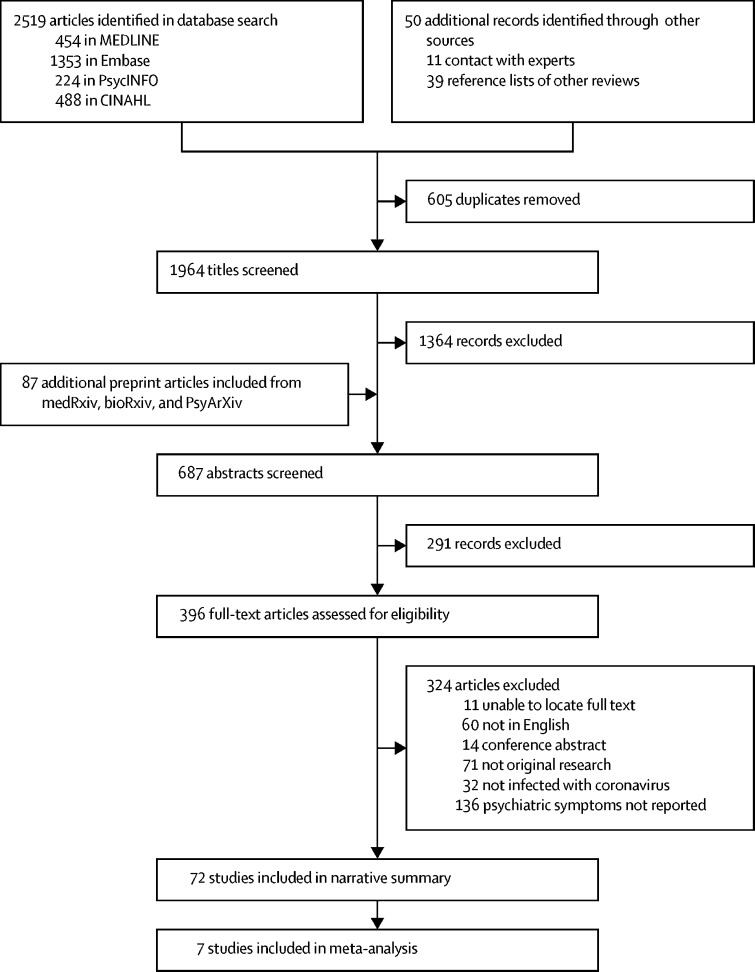
Study selection CINAHL=Cumulative Index to Nursing and Allied Health Literature.

The systematic search identified 1963 studies and 87 preprints, of which 65 independent studies^21^, ^23^, ^33^, ^34^, ^35^, ^36^, ^37^, ^38^, ^39^, ^40^, ^41^, ^42^, ^43^, ^44^, ^45^, ^46^, ^47^, ^48^, ^49^, ^50^, ^51^, ^52^, ^53^, ^54^, ^55^, ^56^, ^57^, ^58^, ^59^, ^60^, ^61^, ^62^, ^63^, ^64^, ^65^, ^66^, ^67^, ^68^, ^69^, ^70^, ^71^, ^72^, ^73^, ^74^, ^75^, ^76^, ^77^, ^78^, ^79^, ^80^, ^81^, ^82^, ^83^, ^84^, ^85^, ^86^, ^87^, ^88^, ^89^, ^90^, ^91^, ^92^, ^93^, ^94^, ^95^ and seven medRxiv preprints^96^, ^97^, ^98^, ^99^, ^100^, ^101^, ^102^ were included in the analyses (figure 1). The number of cases in the included studies ranged from 1 to 997, and the mean age of samples ranged from 12·2 years (SD 4·1) to 68·0 years (single case report). Studies covered China, Canada, France, Hong Kong, Saudi Arabia, South Korea, Japan, Singapore, the UK, and the USA. Several studies had overlapping samples, which made it difficult to estimate the exact number of unique cases identified, although a minimum estimate of total cases was 3559. 47 studies involved SARS-CoV (2068 cases), 13 studies were of MERS-CoV (515 cases), and 12 studies (including seven preprints) described SARS-CoV-2 (976 cases). There were 6390 controls, 2410 of whom were from general population samples used to compare quality-of-life outcomes.

25 studies (table 1) investigated the features of acute SARS (1991 cases) and MERS (489 cases). They include six qualitative studies, two case reports, three case series, one cross-sectional study, one randomised controlled trial, and 12 cohort studies. Two studies^43^, ^73^ systematically assessed signs and symptoms in a representative cohort using a tailored Neuropsychiatric Symptom Checklist, the combined results of which are shown in table 2. During the acute illness, common symptoms among patients admitted to hospital for SARS or MERS included depressed mood (42 [32·6%; 95% CI 24·7–40·9] of 129 patients), anxiety (46 [35·7%; 27·6–44·2] of 129), impaired memory (44 [34·1%; 26·2–42·5] of 129), impaired concentration or attention (39 [38·2%; 29·0–47·9] of 102; in one study), and insomnia (54 [41·9%; 22·5–50·5] of 129). Notably, confusion was reported by 36 (27·9%; 95% CI 20·5–36·0) of 129 patients despite mean ages in the included studies of 37·6 years (SD 12·4) and 41·2 years (18·6). In one study,^33^ 13 (0·7%) of 1744 patients with SARS in Hong Kong were diagnosed with steroid-induced psychotic disorders. In addition, two studies in which disorders were not systematically assessed reported cases of depression (two cases^73^), anxiety disorder (two cases^73^), acute stress reaction (two cases^73^), psychotic depression (one case^33^), psychotic disorder not specified (one case^33^), and deterioration of dementia (one case^73^). Five qualitative studies investigated the experiences of individuals infected with SARS-CoV and MERS-CoV.^34^, ^35^, ^36^, ^37^, ^38^ Loneliness, boredom, and frustration resulting from isolation were prominent.^35^, ^36^, ^37^, ^38^, ^39^ Individuals were often concerned about family members who were already infected, spreading the virus to other acquaintances, and death.^34^, ^36^, ^37^, ^38^ However, two studies noted the enormous gratitude felt by patients for the support they received.^37^, ^38^

**Table 1:** Studies reporting acute psychiatric and neuropsychiatric outcomes of SARS and MERS infections

	**Setting**	**Virus subtype**	**Study design**	**Special population**	**Sample size**	**Age, years**	**Male cases (%)**	**Female cases (%)**	**Outcomes**
Lee et al (2017)^66^	South Korea	MERS-CoV	Case report	..	1 case	68·0	1 (100%)	0	Symptoms: confusion and drowsiness
Schneider et al (2004)^67^	USA	SARS-CoV	Case report	Pregnant	1 case	NR	0	1 (100%)	Symptoms: anxiety
Guery et al (2013)^68^	France	MERS-CoV	Case series	..	2 cases	64·0, 51·0	2 (100%)	0	Symptoms: confusion and disorientation
Cheng et al (2004)^94^	Hong Kong	SARS-CoV	Case series	..	10 cases	Mean 34·8 (SD 15·6)	4 (40%)	6 (60%)	Diagnoses: adjustment disorder, organic hallucinosis, organic manic disorder, and mental disorder not otherwise specified; symptoms: depressed mood, suicidal ideas, anxiety, visual and auditory hallucinations, suspiciousness, persecutory beliefs, delusions of grandeur, elated mood, increased energy, increased activity, and mood swings
Arabi et al (2015)^70^	Saudi Arabia	MERS-CoV	Case series	..	3 cases	Mean 58·7 (SD 6·9)	3 (100%)	0	Symptoms: confusion
Avendano et al (2003)^71^	Toronto, ON, Canada	SARS-CoV	Cohort	Health-care workers	14 cases	Mean 43·9 (SD 10·2)	3 (21%)	11 (79%)	Symptoms: anxiety
Hong et al (2018)^72^	South Korea	MERS-CoV	Cohort	..	30 cases	Mean 49·0 (SD 13·0)	19 (63%)	11 (37%)	Symptoms: altered mental status
Kim et al (2018)^73^	South Korea	MERS-CoV	Cohort	..	27 cases	Mean 41·2 (SD 18·6)	10 (37%)	17 (63%)	Diagnoses: adjustment disorders, depressive disorders, acute stress disorders, delirium, and anxiety disorders; DSM-IV criteria; symptoms: insomnia, depressive mood, tension, disorientation, impaired memory, auditory hallucinations, and aggressive outbursts; scales: PHQ-9, IES-R, PTD-PTNB-PTSS, and KNHANES-short form
Alhumaid et al (2018)^74^	Saudi Arabia	MERS-CoV	Cohort	..	107 cases	Median 54·5 (range 21·0–97·0)	74 (69%)	33 (31%)	Symptoms: confusion
Noorwali et al (2015)^75^	Saudi Arabia	MERS-CoV	Cohort	..	261 cases	Median 47·5 (range 8·0–90·0)	171 (66%)	90 (34%)	Symptoms: altered consciousness
Saad et al (2014)^76^	Saudi Arabia	MERS-CoV	Cohort	..	70 cases	Median 62·0 (range 1·0–90·0)	46 (66%)	24 (34%)	Symptoms: confusion
Mackay et al (2005)^77^	Toronto, ON, Canada	SARS-CoV	Cohort	..	246 cases	NR	95 (39%)	151 (61%)	Symptoms: agitation, confusion, and hallucinations
Sheng et al (2005)^43^	Hong Kong	SARS-CoV	Cohort	..	102 cases	Mean 37·6 (SD 12·4)	35 (34%)	67 (66%)	Scales: NPSC (reporting a broad range of neuropsychiatric symptoms) and GHQ-28
Leung et al (2004)^65^	Hong Kong	SARS-CoV	Cohort	Children	44 cases	Mean 12·2 (SD 4·1)	20 (45%)	24 (55%)	Symptoms: visual hallucinations, auditory hallucinations, impaired attention span, forgetfulness, emotional lability, and depressed mood
Lee et al (2004)^33^	Hong Kong	SARS-CoV	Cohort	..	1744 cases	Mean 32·8 (SD 14·1)	18 (40%)	27 (60%)	Diagnoses: steroid-induced manic episode, steroid-induced psychotic disorder, major depressive episode with psychotic features, and psychotic disorder not otherwise specified
Lau et al (2004)^78^	Hong Kong	SARS-CoV	Cohort	..	88 cases	Mean 42·1 (SD 14·0)	33 (38%)	55 (63%)	Symptoms: confusion, anxiety, and depression
Chua et al (2004)^79^	Hong Kong	SARS-CoV	Cohort	..	79 cases; 145 controls	34·0 (estimated)	27 (34%)	52 (66%)	Scales: PSS-10
Jeong et al (2016)^80^	South Korea	MERS-CoV	Cross-sectional	..	36 cases; 1656 controls	Mean 52·3 (SD 15·0)	18 (50%)	18 (50%)	Scales: STAXI and GAD-7
Koller et al (2006)^35^	Toronto, ON, Canada	SARS-CoV	Qualitative	Children	5 cases	NR	NR	NR	Qualitative: sadness, worry, and fear
Almutairi et al (2018)^34^	Saudi Arabia	MERS-CoV	Qualitative	Health-care workers	7 cases	Mean 47·0 (SD 15·9)	3 (43%)	4 (57%)	Qualitative: anxiety, fear, and despair
Mok et al (2005)^37^	Hong Kong	SARS-CoV	Qualitative	Nurses	10 cases	Range 20·0–47·0	2 (20%)	8 (80%)	Qualitative: uncertainty, guilt, fear of death, isolation, and loneliness
Tiwari et al (2003)^38^	Hong Kong	SARS-CoV	Qualitative	..	12 cases	NR	NR	NR	Qualitative: fear and frustration
Li et al (2004)^39^	Hong Kong	SARS-CoV	Qualitative	Children	4 cases	Range 7·0–13·0	2 (50%)	2 (50%)	Qualitative: social isolation; symptoms: psychological distress
Maunder et al (2003)^36^	Toronto, ON, Canada	SARS-CoV	Qualitative	..	19 cases	NR	NR	NR	Symptoms: insomnia, anxiety, and exacerbation of a panic disorder
Loutfy et al (2003)^81^	Toronto, ON, Canada	SARS-CoV	Randomised controlled trial treated as a cohort study	..	22 cases	Median 48·0 (range 27·0–56·0)	6 (27%)	16 (73%)	No depression with interferon alfacon-1 treatment

Proportions might not sum to 100% as a result of rounding. GAD-7=Generalised Anxiety Disorder-7. GHQ-28=General Health Questionnaire-28. IES-R=Impact of Event Scale Revised. KNHANES=Korea National Health and Nutrition Examination Survey. MERS-CoV=Middle East respiratory syndrome coronavirus. NPSC=Neuropsychiatric Symptom Checklist. NR=not reported. PHQ-9=Patient Health Questionnaire-9. PSS-10=Perceived Stress Scale-10. PTD-PTNB-PTSS=Peri-Traumatic Dissociation–Post-Traumatic Negative Beliefs–Post-Traumatic Social Support scale. SARS-CoV=severe acute respiratory syndrome coronavirus. SARS-CoV-2=severe acute respiratory syndrome coronavirus 2. STAXI=State-Trait Anger Expression Inventory.

**Table 2:** Prevalence of psychiatric and neuropsychiatric signs and symptoms reported by acute and post-illness studies that used systematic assessments^39^, ^43^, ^46^, ^48^, ^54^, ^73^, ^83^, ^86^, ^92^, ^93^

	**Acute**				**Post-illness**			
	Studies	Cases	Sample size	Prevalence (95% CI)	Studies	Cases	Sample size	Prevalence (95% CI)
Any	1	17	27	63·0% (43·8–80·4)	1	0	4	0 (0·0–39·1)
Insomnia	2	54	129	41·9% (22·5–50·5)	4	34	280	12·1% (8·6–16·3)
Anxiety	2	46	129	35·7% (27·6–44·2)	2	21	171	12·3% (7·7–17·7)
Impaired concentration or attention	1	39	102	38·2% (29·0–47·9)	2	34	171	19·9% (14·2–26·2)
Impaired memory	2	44	129	34·1% (26·2–42·5)	3	44	233	18·9% (14·1–24·2)
Depressed mood	2	42	129	32·6% (24·7–40·9)	5	35	332	10·5% (7·5–14·1)
Confusion	2	36	129	27·9% (20·5–36·0)	1	1	621	0·2% (0·0–0·7)
Emotional lability	1	30	102	29·4% (0·4–7·3)	1	24	102	23·5% (15·8–32·3)
Altered consciousness	1	17	82	20·7% (12·6–30·3)	NA	NA	NA	NA
Pressured speech	1	21	102	20·6% (13·3–29·0)	1	12	102	11·8% (6·1–18·8)
Euphoria	1	8	102	7·8% (3·3–14·0)	1	11	102	10·8% (5·4–17·6)
Aggression	1	2	27	7·4% (0·2–21·1)	1	1	102	1·0% (0·0–4·2)
Irritability	1	5	102	4·9% (1·4–10·1)	3	28	218	12·8% (8·7–17·6)
Auditory hallucinations	2	6	129	4·7% (1·6–9·1)	1	1	102	1·0% (0·0–4·2)
Persecutory ideas	1	4	102	3·9% (0·9–8·7)	1	2	102	2·0% (0·0–5·8)
Visual hallucinations	1	2	102	2·0% (0·0–5·8)	NA	NA	NA	NA
Suicidality	1	2	102	2·0% (0·0–5·8)	1	0	102	0 (0·0–1·7)
Fatigue	NA	NA	NA	NA	4	61	316	19·3% (15·1–23·9)
Frequent recall of traumatic memories	NA	NA	NA	NA	1	55	181	30·4% (23·9–37·3)
Sleep disorder	NA	NA	NA	NA	1	14	14	100% (88·0–100·0)
Psychotic symptoms (unspecified)	NA	NA	NA	NA	1	4	90	4·4% (1·0–9·9)
Self-harm	NA	NA	NA	NA	1	1	102	1·0% (0·0–4·2)

NA=not available.

40 studies investigated psychiatric features after the initial infection had resolved (table 3). 35 studies describe 1192 SARS survivors and five studies describe 140 MERS survivors. They include six qualitative studies, one case report, one case series, six cross-sectional studies, and 26 cohort studies. Follow-up duration varied from 60 days to 12 years. In the post-illness stage, depressed mood (35 [10·5%; 95% CI 7·5–14·1] of 332 patients), euphoria (11 [10·8%; 5·4–17·6] of 102; in one study), pressured speech (12 [11·8%; 6·1–18·8] of 102; in one study), insomnia (34 [12·1%; 8·6–16·3] of 280), anxiety (21 [12·3%; 7·7–17·7] of 171), irritability (28 [12·8%; 8·7–17·6] of 218), memory impairment (44 [18·9%; 14·1–24·2] of 233), fatigue (61 [19·3%; 15·1–23·9] of 316), emotional lability (24 [23·5%; 5·8–32·3] of 102; in one study), traumatic memories (55 [30·4%; 23·9–37·3] of 181; in one study), and sleep disorder (14 [100·0%; 88·0–100·0] of 14; in one study) were frequently reported (table 2). Four studies assessed factors associated with psychiatric outcomes following SARS and are summarised in table 4.^41^, ^42^, ^44^, ^46^ Six qualitative papers discussed the longer-term outcomes for patients with SARS.^21^, ^34^, ^37^, ^38^, ^47^, ^48^ A major theme was the stigma that patients experienced, including from health-care professionals who did not believe their more chronic symptoms, institutions, the general public, or even their own families, friends, and colleagues.^21^, ^34^, ^37^, ^47^, ^48^ However, two studies discussed positive psychological outcomes, with patients gaining a better perspective on life and valuing their relationships, health, and everyday existence more.^37^, ^38^

**Table 3:** Studies reporting post-illness psychiatric and neuropsychiatric outcomes of SARS-CoV and MERS-CoV infections

	**Setting**	**Virus subtype**	**Study design**	**Follow-up timepoint**	**Special population**	**Sample size**	Mean (SD)*age, years	**Male cases (%)**	**Female cases (%)**	**Outcomes**
Schneider et al (2004)^67^	USA	SARS-CoV	Case report	3 months	Pregnancy	1 case	NR	0	1	Symptoms: anxiety
Cheng et al (2006)^45^	Hong Kong	SARS-CoV	Case series	2, 3, 4, 5, and 6 months after discharge	..	57 cases	38·1 (10·4)	19 (33%)	38 (67%)	Scales: BDI, BAI (SARS Appraisal Inventory), and Thriving Scale
Hui et al (2005)^82^	Hong Kong	SARS-CoV	Cohort	3 months, 6 months, and 12 months	..	97 cases; 1939 controls	36·9 (9·5)	39 (40%)	58 (60%)	Scales SF-36 (and subscales)
Mak et al (2009)^40^	Hong Kong	SARS-CoV	Cohort	18 months	..	143 cases	38·4 (12·4)	53 (37%)	90 (63%)	Scale: SF-36
Wing et al (2012)^46^	Hong Kong	SARS-CoV	Cohort	Mean 39 months (SD NR)	..	181 cases	No psychiatric disorder: 44·9 (15·6); lifetime or current: 44·5 (12·0); current psychiatric condition: 45·6 (12·0)	57 (31%)	124 (69%)	Symptoms: fatigue, frequent recall of SARS memories; diagnoses: chronic fatigue syndrome, major depressive disorder, post-traumatic stress disorder, somatoform pain disorder, panic disorder; symptoms: fatigue, and intrusive memories; scales: HADS, IES, GAF, WHOQOL (and subscales), and WSAS (implied)
Han et al (2003)^83^	Guandong, China	SARS-CoV	Cohort	Mean 59·7 days (SD 22·8)	..	69 cases	NR	29 (42%)	40 (58%)	Symptoms: insomnia, vexation, low spirit, fear, poor concentration, poor memory, and feelings of guilt
Lam et al (2006)^55^	Hong Kong	SARS-CoV	Cohort	Mean 60·0 days (SD 23·9)	..	116 cases	45·6 (15·1)	51 (44%)	65 (56%)	Scale: SF-36
Guo et al (2019)^58^	Guandong, China	SARS-CoV	Cohort	12 years	..	67 cases	Data from original cohort only	Data from original cohort only	−	Scale: SF-36
Lee et al (2019)^54^	South Korea	MERS-CoV	Cohort	12 months and 18 months	..	52 cases	49·7 (12·0)	32 (62%)	20 (38%)	Scales: PHQ-9, FSS, and IES-R
Mak et al (2010)^41^	Hong Kong	SARS-CoV	Cohort	30 months	..	90 cases	No post-traumatic stress disorder: 40·5 (11·6); post-traumatic stress disorder: 42·8 (13·4)	34 (38%)	56 (62%)	Diagnoses: post-traumatic stress disorder; scales: FIC, CWCQ, and MHLC
Yoon et al (2016)^84^	South Korea	MERS-CoV	Cohort	NR	..	62 cases	NR	NR	NR	Other: referral for outpatient psychiatric treatment
Moldofsky et al (2011)^85^	Toronto, ON, Canada	SARS-CoV	Cohort	Mean 19·8 months (range 13–36)	Individuals unable to return to former occupation; mainly health-care workers	22 cases; 21 fibromyalgia controls, 7 healthy controls	46·3 (11·0)	3 (14%)	19 (86%)	Scales: BDI, PCL-C, SAQ, and WPSI
Lam et al (2009)^49^	Hong Kong	SARS-CoV	Cohort	Mean 41·3 months (range 31–51)	..	233 cases	43·3 (13·7)	69 (30%)	164 (70%)	Diagnoses: any psychiatric illness, post-traumatic stress disorder, depression, somatoform pain disorder, panic disorder, obsessive compulsive disorder, and chronic fatigue syndrome. Scales: HADS, IES-R, and CFQ
Hong et al (2009)^56^	Beijing, China	SARS-CoV	Cohort	Mean 53 days (SD 31), 7 months, 10 months, 20 months, and 46 months	..	70 cases	38·5 (12·3)	23 (33%)	47 (67%)	Diagnoses: post-traumatic stress disorder; scales: IES, SAS, SCL-90, SDS, SDSS, and SF-36
Mak et al (2009)^86^	Hong Kong	SARS-CoV	Cohort	30 months	..	90 cases;1394 controls (Hong Kong normative data)	41·1 (12·1)	56 (62%)	34 (38%)	Symptoms: psychotic symptoms; diagnoses: any psychiatric disorder, post-traumatic stress disorder, anxiety disorders (and subtypes), depression (and subtypes), and substance misuse; scale: IES-R, HADS, and SF-36
Bonanno et al (2008)^42^	Hong Kong	SARS-CoV	Cohort	6 months, 12, months, and 18 months	..	997 cases, 2410 controls (Hong Kong normative data)	42·0 (14·0)	389 (39%)	608 (61%)	Scale: SF-12
Lee et al (2007)^53^	Hong Kong	SARS-CoV	Cohort	12 months	..	Two overlapping samples of 79 and 96 cases, and 145 and 112 controls	Stratified across group, year, and age range	62 (35%)	113 (65%)	Scales: PSS-10 (reported in 1386 participants), DASS-21, GHQ-12, and IES-R
Tansey et al (2007)^59^	Toronto, ON, Canada	SARS-CoV	Cohort	3 months, 6, months, and 12 months	..	117 cases	Median 42·0 (range 33·0–51·0)	39 (33%)	78 (67%)	Scale: SF-36
Lau et al (2005)^87^	Hong Kong	SARS-CoV	Cohort	About 2 months from onset of illness	..	15 cases	35·0 (10·9)	8 (53%)	7 (47%)	Diagnoses: anxiety depression, and steroid psychosis; scale: WHOQOL
Leow et al (2005)^88^	Singapore	SARS-CoV	Cohort	3 months after recovery	..	61 cases	Median 36·5 (range 25·5-47·5)	14 (23%)	47 (77%)	Symptoms: fatigue
Wu et al (2005)^52^	Hong Kong	SARS-CoV	Cohort	1 month and 3 months after discharge	..	131 cases	41·8 (14·0)	57 (44%)	74 (56%)	Scales: IES and HADS
Sheng et al (2005)^43^	Hong Kong	SARS-CoV	Cohort	Mean 42 days (range 26–86) after discharge	..	102 cases	37·6 (12·4)	35 (34%)	67 (66%)	Symptoms: numerous neuropsychiatric symptoms from NPSC; scales: NPSC and GHQ-28
Cheng et al (2004)^69^	Hong Kong	SARS-CoV	Cohort	1 month after recovery	..	100 cases; 184 controls	37·1 (12·1)	34 (34%)	66 (66%)	Scales: RSES, GHQ-28, and WHOQOL-BREF
Cheng et al (2004)^44^	Hong Kong	SARS-CoV	Cohort	At least 4 weeks after discharge; mean 43·8 days (SD 13·6)	..	180 cases;649 healthy controls and 189 psychiatric outpatient controls	36·9 (11·1)	60 (33%)	120 (67%)	Scales: BAI, BDI, and SIS
Ngai et al (2010)^60^	Hong Kong	SARS-CoV	Cohort	3 months, 6 months, 12 months, 18 months, and 24 months	..	55 cases and 538 controls (Hong Kong normative data)	44·4 (13·2)	19 (35%)	36 (65%)	Scale: SF-36
Hui et al (2005)^89^	Hong Kong	SARS-CoV	Cohort	3 months and 6 months	..	110 cases	35·6 (9·8)	44 (40%)	66 (60%)	Scale: SF-36
Lau et al (2005)^90^	Hong Kong	SARS-CoV	Cohort	2 weeks after discharge	..	171 cases and 2410 controls (Hong Kong normative data)	37·4 (12·7)	60 (35%)	111 (65%)	Scale: SF-36
Li et al (2006)^91^	Hong Kong	SARS-CoV	Cohort	3 months, 6 months, and 12 months	ICU admission with acute respiratory distress syndrome	59 cases	47·0 (16·0)	34 (58%)	25 (42%)	Scale: SF-36
Lau et al (2005)^95^	Hong-Kong	SARS-CoV	Randomised controlled trial treated as a cohort study	At least 8 weeks after discharge	Subnormal exercise tolerance	133 cases	37·0 (10·2)	45 (34%)	88 (66%)	Scale: SF-36
Tso et al (2004)^92^	Hong Kong	SARS-CoV	Cross-sectional	Median 6·6 weeks (SD 1·1) after onset	..	62 cases	37·1 (13·0)	28 (45%)	34 (55%)	Symptoms: forgetfulness, depression, and insomnia
Lo et al (2005)^93^	Singapore	SARS-CoV	Cross-sectional	6 months	..	14 cases; 30 controls	Range 20–48	2 (14%)	12 (86%)	Symptoms: fatigue and sleep disturbance
Wu et al (2005)^50^	Hong Kong	SARS-CoV	Cross-sectional	1 month	..	195 cases	41·5 (14·0)	84 (43%)	111 (57%)	Scales: IES-R and HADS
Kwek et al (2006)^51^	Singapore	SARS-CoV	Cross-sectional	6 weeks and 12 weeks	..	63 cases; Singapore normative data as control	34·8 (10·5)	13 (21%)	50 (79%)	Scales: IES, HADS, and SF-36
Batawi et al (2019)^57^	Saudi Arabia	MERS-CoV	Cross-sectional	Mean 13·8 months (SD 3·4)	..	78 cases; 57 controls (non-MERS-CoV severe acute respiratory infection)	45·0 (13·0)	56 (72%)	22 (28%)	Scale: SF-36
Jeong et al (2016)^80^	Seoul, Gyeonggi, Chungcheong, and Gangwon, South Korea	MERS-CoV	Cross-sectional	4, 5, and 6 months after isolation	..	36 cases;1656 controls without MERS who had also been isolated	52·3 (15·0)	18 (50%)	18 (50%)	Scales: STAXI and GAD-7
Almutairi et al (2018)^34^	Saudi Arabia	MERS-CoV	Qualitative	NR	Health-care workers	7 cases	42·0 (16·2)	3 (43%)	4 (57%)	Qualitative: stigma and underestimation of illness severity
Siu (2016)^47^	Hong Kong	SARS-CoV	Qualitative	NR	Individuals practising tai chi	35 cases	Range 38–69	13 (37%)	22 (63%)	Qualitative: emotional suffering, stigma, and passivity
Siu (2008)^21^	Hong Kong	SARS-CoV	Qualitative	NR	..	30 cases	NR	NR	NR	Qualitative: stigma
Mok et al (2005)^37^	Hong Kong	SARS-CoV	Qualitative	NR	Nurses	10 cases	Range 20–47	2 (20%)	8 (80%)	Themes: anger, guilt, unpreparedness, fear, isolation, physical symptoms, support, and changing perspective
Lee et al (2005)^48^	Hong Kong	SARS-CoV	Qualitative	NR	..	47 cases; 852 controls (neighbouring residents)	Only reported for entire cohort including non-infected	Only reported for entire cohort including non-infected	..	Symptoms: insomnia, irritability, and low mood
Li et al (2004)^39^	Hong Kong	SARS-CoV	Qualitative	5 months after discharge	Children	4 cases	Range 7–13	2 (50%)	2 (50%)	Symptoms: psychological distress

Proportions might not sum to 100% as a result of rounding. BAI=Beck Anxiety Inventory. BDI=Beck Depression Inventory. CFQ=Cognitive Failures Questionnaire. CWCQ=Chinese Ways of Coping Questionnaire. DASS-21=Depression, Anxiety and Stress Scale 21 items. FIC=Functional Impairment Checklist. FSS=Fatigue Severity Scale. GAD-7=Generalised Anxiety Disorder-7. GAF=Global Assessment of Functioning. GHQ-12=General Health Questionnaire-12. GHQ-28=General Health Questionnaire-28. HADS=Hospital Anxiety and Depression Scale. ICU=intensive care unit. IES=Impact of Event Scale. IES-R=Impact of Event Scale Revised. MERS-CoV=Middle East respiratory syndrome coronavirus. MHLC=Multidimensional Health Locus of Control. NPSC=Neuropsychiatric Symptom Checklist. NR=not reported. PCL-C=PTSD Checklist, Civilian Version. PHQ-9=Patient Health Questionnaire-9. PSS-10=Perceived Stress Scale 10. RSES=Rosenberg Self-Esteem Scale. SAQ=Sleep Assessment Questionnaire. SARS-CoV=severe acute respiratory syndrome coronavirus. SAS=Zung Self-Rating Anxiety Scale. SCL-90=Symptom Checklist 90. SDS=Zung Self-Rating Depression Scale. SDSS=Social Disability Screening Schedule. SF-12=Short Form 12 Health Survey Questionnaire. SF-36=Short Form 36 Health Survey Questionnaire. SIS=SARS Impact Scale. STAXI=State-Trait Anger Expression Inventory. WHOQOL=WHO Quality of Life. WPSI=Wahler Physical Symptom Inventory. WSAS=Work and Social Adjustment Scale.

* Data are mean (SD) unless otherwise stated.

**Table 4:** Factors associated with psychiatric and neuropsychiatric outcomes in SARS

	**Outcome**	**Result**
**Demographic**		
Female sex	Post-traumatic stress disorder diagnosis (DSM-IV)	OR 3·85 (95% CI 1·18–12·54)^41^
Female sex	Chronic illness compared with resilience (based on SF-12)	OR 2·17 (p<0·01)*^42^
Female sex	Moderate or severe range score on the BAI or BDI	OR 1·8 (95% CI 0·9–3·6)^44^
Female sex	Current psychiatric disorder (DSM-IV)	OR 2·0 (95% CI 1·03–3·89)^46^
Age	Chronic illness compared to resilience (based on SF-12)	OR 1·01 (not significant)*^42^
Health-care worker	Moderate or severe range score on the BAI or BDI	OR 3·8 (95% CI 1·8–8·2)^44^
Health-care worker	Current psychiatric disorder (DSM-IV)	OR 2·59 (95% CI 1·38–4·87)^46^
Health-care worker	Post-traumatic stress disorder diagnosis (DSM-IV)	OR 2·92 (95% CI 1·08–7·88)^41^
Married	Current psychiatric disorder (DSM-IV)	OR 1·14 (0·60–2·18)^46^
**Baseline illness**		
Previous chronic physical illness	Post-traumatic stress disorder diagnosis (DSM-IV)	OR 4·38 (95% CI 1·06–18·02)^41^
Previous chronic physical illness	Moderate or severe range score on the BAI or BDI	OR 0·8 (95% CI 0·3–2·4)^44^
**Disease-related**		
Presence of avascular necrosis	Post-traumatic stress disorder diagnosis (DSM-IV)	OR 2·91 (95% CI 1·06–8·02)^41^
Functional Impairment Checklist, disability score	Post-traumatic stress disorder diagnosis (DSM-IV)	OR 2·44 (95% CI 1·66–3·56)^41^
Average pain	Post-traumatic stress disorder diagnosis (DSM-IV)	OR 1·69 (95% CI 1·31–2·19)^41^
Distressing pain after SARS	Post-traumatic stress disorder diagnosis (DSM-IV)	OR 36·01 (95% CI 2·10–617·59)^41^
**Psychological**		
SARS-related worry	Chronic illness compared to resilience (based on SF-12)	OR 1·04 (p<0·05)*^42^
Chance locus of control (Multidimensional Health Locus of Control scale)	Post-traumatic stress disorder diagnosis (DSM-IV)	OR 1·22 (95% CI 1·09–1·37)^41^
Frequent recall of SARS memories	Current psychiatric disorder (DSM-IV)	OR 13·5 (95% CI 6·2–29·4)^46^
**Social**		
Social network size	Chronic illness compared to resilience (based on SF-12)	OR 0·99 (not significant)*^42^
Death of relative due to SARS	Moderate or severe range score on the BAI or BDI	OR 3·4 (95% CI 1·0–12·2)^44^
Medicolegal involvement	Current psychiatric disorder (DSM-IV)	OR 7·69 (95% CI 2·15–27·6)^46^

Unadjusted ORs were reported, except for the outcomes marked. BAI=Beck Anxiety Inventory. BDI=Beck Depression Inventory. OR=odds ratio. SARS=severe acute respiratory syndrome. SF-12=Short Form 12 Health Survey Questionnaire.

* Only adjusted ORs were available; 95% CIs were not available.

**Figure 2:**
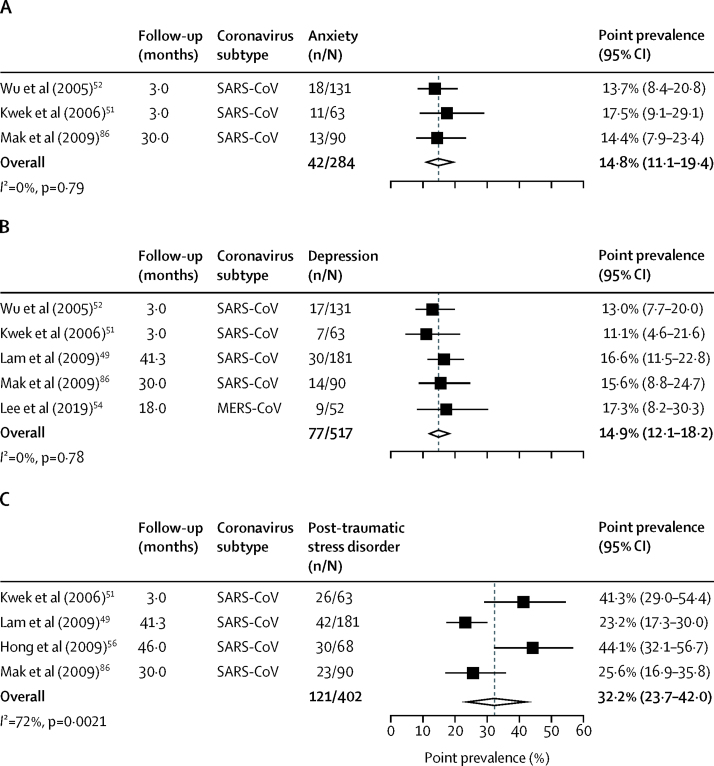
Forest plots of pooled prevalence of anxiety (A), depression (B), and post-traumatic stress disorder (C) in individuals who recovered from coronavirus infection MERS-CoV=Middle East respiratory syndrome coronavirus. SARS-CoV=severe acute respiratory syndrome coronavirus.

In the post-illness phase, the point prevalence of anxiety disorder diagnoses was 14·8% (95% CI 11·1–19·4; 42 of 284 cases from three studies; figure 2A) at a mean follow-up of 11·6 months (SD 12·6). The point prevalence of depression was 14·9% (95% CI 12·1–18·2; 77 of 517 cases from five studies; figure 2B) at a mean follow-up of 22·6 months (SD 16·7). The point prevalence of post-traumatic stress disorder was 32·2% (95% CI 23·7–42·0; 121 of 402 cases from four studies; figure 2C) at mean follow-up of 33·6 months (SD 14·2). Point prevalences were used in all studies except in one study,^49^ in which it was not clear whether the value was in fact an estimate of period prevalence.

For symptom severity scores, standardised mean differences could not be generated because control groups were not used in included studies. Hence, studies using different symptom scales to assess the same symptoms (eg, Hospital Anxiety and Depression Scale [HADS] and Beck Depression Inventory [BDI]) could not be combined. The weighted mean symptom score for the HADS anxiety subscale, with a clinical cutoff of 8, was 6·5 (95% CI 3·9–9·1; assessed in 364 cases from three studies; appendix p 12).^49^, ^50^, ^51^, ^52^ The weighted mean symptom score for the HADS depression subscale, with a clinical cutoff of 8, was 6·2 (95% CI 3·7–8·6; 364 cases from three studies).^49^, ^50^, ^51^ The weighted mean symptom score was 10·8 (95% CI 6·9–14·7; 397 cases from three studies) for the Impact of Event Scale Revised (IES-R) Intrusion subscale,^49^, ^50^, ^51^, ^52^, ^53^ 8·8 (5·0–12·5; 397 cases from three studies) for the IES-R Avoidance subscale,^49^, ^50^, ^51^, ^53^ 8·1 (5·1–11·1; 397 cases from three studies) for the IES-R Hyperarousal subscale,^49^, ^50^, ^52^, ^53^ and 20·7 (7·8–33·5; 115 cases across two studies) for IES-R Total (clinical cutoff of 24)^51^, ^54^ at a mean follow-up time of 9·8 months (SD 10·6).

Health-related quality of life was lower in patients after SARS-CoV infection across the three mental health-related subscales of the Short Form 36 Health Survey Questionnaire (SF-36; range 0–100 points) than among the general population sample obtained using a telephone survey with an unknown response rate.^103^, ^104^ The pooled mean difference was −26·4 points (95% CI −37·0 to −15·7, p<0·0001; 187 cases from two studies) for social functioning, −15·4 (−31·2 to 0·5, p=0·057; 187 cases from two studies) for role limitation due to emotional problems, and −10·6 (−13·9 to −7·4, p<0·0001; 187 cases from two studies) for the mental health subscale at a mean follow-up time of 20·7 months (SD 9·0; appendix p 11). When combined with data from studies of SARS and MERS that did not have data from a control group,^40^, ^51^, ^55^, ^56^, ^57^ the weighted mean SF-36 scores were 68·1 (95% CI 60·1–76·0; assessed in 581 cases from 11 studies) for social functioning, 44·1 (43·0–45·2) for role limitation due to emotional problems, and 52·0 (51·2–52·8) for the mental health subscale (appendix p 13). With regard to employment, 446 (76·9%; 95% CI 68·1–84·6) of 580 patients from six studies had returned to work at a mean follow-up time of 35·3 months (SD 40·1; appendix p 15).^46^, ^49^, ^58^, ^59^, ^60^ The proportion of patients who were admitted to an ICU or ventilated are presented in the appendix (p 16). Results of heterogeneity and sensitivity analyses can be seen in the appendix (p 17).

12 studies (including seven preprints) described the features of 976 patients with acute SARS-CoV-2 infection (table 5). Seven studies (including four preprints) were from Wuhan and reported data from at least 575 unique cases. Another three preprints described 343 cases from Chongqing and Zhejiang in China, and Hong Kong. Two preprints used rating scales to systematically assess depressive and anxiety symptoms.^96^, ^97^ In one study,^96^ 50 (35%) of 144 patients had symptoms of anxiety and 41 (28%) had symptoms of depression, although these assessments were not diagnostic. In the other study,^97^ 26 patients with SARS-CoV-2 infection were compared with patients with other forms of pneumonia and age-matched and sex-matched healthy controls; scores on both the Hamilton Depression Scale and the Hamilton Anxiety Scale were higher for the SARS-CoV-2 group than for either of the other groups, but these scores improved significantly after the first week of their hospital stay.

**Table 5:** Studies reporting acute psychiatric and neuropsychiatric outcomes of SARS-CoV-2 infections

	**Preprint**	**Setting**	**Virus subtype**	**Study design**	**Special population**	**Sample size**	Mean (SD)*age, years	**Male cases (%)**	**Females cases (%)**	**Outcomes**
Moriguchi et al (2020)^64^	No	Japan	SARS-CoV-2	Case report	..	1 case	24 (NR)	1 (100%)	0	Symptom: impaired consciousness; diagnosis: meningitis-encephalitis
Helms et al (2020)^61^	No	France	SARS-CoV-2	Case series	ICU admissions	58 cases	NR	NR	NR	Symptoms: agitation, confusion, inattention, disorientation, and poorly organised movements in response to command; diagnoses: dysexcutive syndrome and encephalopathy; investigations: MRI brain, EEG, and CSF analysis
Chen et al (2020)^63^	No	Wuhan, China	SARS-CoV-2	Cohort	..	99 cases	55·5 (13·1)	67 (68%)	32 (32%)	Symptom: confusion
Chen et al (2020)^62^	No	Wuhan, China	SARS-CoV-2	Cohort	..	21 cases	Median 56·0 (IQR 50·0–65·0)	17 (81%)	4 (19%)	Symptom: coma; diagnosis: hypoxic encephalopathy
Zhang et al (2020)^98^	Yes	Wuhan, China	SARS-CoV-2	Cohort	Deaths	82 cases	Median 72·5 (IQR 65·0–80·0)	54 (66%)	28 (34%)	Symptom: consciousness problem
Qi et al (2020)^100^	Yes	Chongqing, China	SARS-CoV-2	Cohort	..	267 cases	Median 48·0 (IQR 35·0–65·0)	149 (56%)	118 (44%)	Symptom: confusion
Huang et al (2020)^99^	Yes	Wuhan, China	SARS-CoV-2	Cohort	Deaths	36 cases	69·2 (9·6)	25 (69%)	11 (31%)	Symptom: disturbance of consciousness
Mao et al (2020)^23^	No	Wuhan, China	SARS-CoV-2	Cohort	..	214 cases	52·7 (15·5)	87 (40%)	127 (60%)	Symptom: impaired consciousness
Leung et al (2020)^101^	Yes	Hong Kong	SARS-CoV-2	Cohort	..	50 cases	55·2 (19·5)	23 (46%)	27 (54%)	Symptom: confusion
Fu et al (2020)^102^	Yes	Wuhan, China	SARS-CoV-2	Cohort	..	50 cases	Median 64·0 (IQR 37·0–87·0)	27 (54%)	23 (46%)	Symptom: insomnia
Yang et al (2020)^97^	Yes	Zhejiang, China	SARS-CoV-2	Cohort	..	26 cases, 87 pneumonia controls, 30 healthy controls	Mean 56·0 (range 27·0–86·0)	9 (35%)	17 (65%)	Scales: HAMD and HAMA
Kong et al (2020)^96^	Yes	Wuhan, China	SARS-CoV-2	Cross-sectional	..	144 cases	50·0 (13·7)	70 (49%)	74 (51%)	Scales: HADS and PSSS

CSF=cerebrospinal fluid. EEG=electroencephalogram. HADS=Hospital Anxiety and Depression Scale. HAMA=Hamilton Anxiety Scale. HAMD=Hamilton Depression Scale. ICU=intensive care unit. NR=not reported. PSSS=Perceived Social Support Scale. SARS-CoV-2=severe acute respiratory syndrome coronavirus 2.

* Data are mean (SD) unless otherwise stated.

A recently published study^61^ of 58 patients with COVID-19 who had been admitted to two ICUs in France described agitation in 40 (69%) patients after withdrawal of sedation and neuromuscular blockade. It also reported confusion in 26 (65%) of 40 patients who were assessed using the Confusion Assessment Method for the ICU. Some patients had neuropsychiatric investigations including brain MRI (13 [22%] of 58 patients), electroencephalogram (EEG; eight [14%] patients), and lumbar puncture (seven [12%] patients). MRI demonstrated larger leptomeningeal spaces in eight (62%) of 13 patients as well as two recent asymptomatic ischaemic strokes. EEG changes were non-specific, with diffuse bifrontal slowing consistent with encephalopathy described in one of eight patients. Of seven patients who had lumbar puncture, cerebrospinal fluid analysis identified oligoclonal bands in two patients and elevated protein and IgG in another. At discharge, 15 (33%) of 45 patients who were assessed had a dysexecutive syndrome with symptoms such as inattention, disorientation, or poorly organised movements in response to command.^61^

The only other systematic assessment of neuropsychiatric presentations was from a preprint^98^ that found altered consciousness to be present in 17 (21%) of 82 patients with COVID-19 who subsequently died. Overall, altered consciousness or encephalopathy was reported in five studies.^23^, ^62^, ^98^, ^99^ Four other studies (two preprints) reported cases of confusion or disturbance of consciousness, although not systematically, with prevalence ranging between 2·0% (95% CI 0·4–10·5) and 22·2% (11·7–38·1).^63^, ^99^, ^100^, ^101^ In terms of neuropsychiatric features of specific neurological consequences of SARS-CoV-2 infection, there was one report of meningitis-encephalitis and two cases where hypoxic encephalopathy was specified in peer-reviewed studies.^62^, ^64^

Overall, for the 65 peer-reviewed studies, 32 were deemed to be of low quality, 30 were deemed to be of moderate quality, and three were deemed to be of high quality. Two preprints were of low quality, four moderate quality, and one was high quality. Across studies, the main weaknesses were due to limited assessment of pre-infection psychiatric symptoms and the lack of adequate comparison groups. Results of the study quality assessment are described in the appendix (pp 18–20).

## Discussion

To our knowledge, this is the first systematic review and meta-analysis of the psychiatric consequences of coronavirus infection. We identified 72 independent studies that provided data on both the acute and post-illness psychiatric and neuropsychiatric features of coronavirus infection, including seven medRxiv preprints. The scientific literature predominantly consists of data on patients with SARS and MERS treated in hospital, so there should be caution in generalising any findings to COVID-19, particularly for patients who have mild symptoms. Our main findings are that signs suggestive of delirium are common in the acute stage of SARS, MERS, and COVID-19; there is evidence of depression, anxiety, fatigue, and post-traumatic stress disorder in the post-illness stage of previous coronavirus epidemics, but there are few data yet on COVID-19.

In SARS and MERS in the acute stage, using data from two studies, the most important finding was that confusion occurred in 27·9% of patients, suggesting that delirium was common. Other common psychiatric findings were depression, anxiety, and insomnia. Diagnoses of mania and psychosis did occur in a small minority (0·7%), but in a small sample this diagnosis appeared to be almost entirely related to use of exogenous corticosteroids, which are rarely prescribed to treat SARS-CoV-2 infection. Notably, insomnia, emotional lability, irritability, pressured speech, and euphoria were relatively common, suggesting that although a full syndrome of mania was uncommon, subthreshold symptoms might be present.

In SARS and MERS, after recovery from the infection, sleep disorder, frequent recall of traumatic memories, emotional lability, impaired concentration, fatigue, and impaired memory were reported in more than 15% of patients at a follow-up period ranging between 6 weeks and 39 months. Emotional lability, pressured speech, and euphoria were only reported by patients and relatives after a short follow-up (mean 42 days [range 26–86]) in one study^43^ in which corticosteroids had frequently been prescribed at high doses and symptoms; therefore, it might be of limited relevance to the COVID-19 pandemic. The point prevalences of anxiety disorders, depression, and post-traumatic stress disorder were high, although the lack of adequate comparison groups or assessment of previous psychiatric disorder means that it is hard to separate the effects of the infection from the impact of an epidemic on the population as a whole or the possibility that selection bias led to the high prevalence figures. In terms of severity, mean scores for depression and anxiety on standard scales were below clinical cutoffs. Measures of health-related quality of life were considerably lower in patients with SARS than in control groups. However, the impairment in social functioning was greater than the effects on mental health (appendix p 11), suggesting that the effect of coronaviruses is broad and not specific to mental health. Some positive effects in terms of personal growth during adversity were noted.

In terms of applicability to COVID-19, conclusions must be cautious because data on the acute effects of the illness are limited and no data exist on the post-illness phase, and the higher mortality of SARS and MERS might be correlated with poorer psychiatric outcomes.^11^, ^105^ The information available suggests that in the acute stage—as in SARS and MERS—confusion is a common feature, so delirium is probably a significant clinical problem. In the longer term, the data from SARS and MERS suggest that the prevalence of depression, anxiety, post-traumatic stress disorder, and fatigue might be high, but as yet data on these diagnoses in patients with COVID-19 are preliminary or unpublished. In patients with severe illness requiring ICU admission, neurocognitive impairment might be a feature. We found only three cases of SARS-CoV-2-related psychiatric symptoms that were explicitly linked to hypoxic or encephalitic brain injury; this finding is consistent with the rarity of case reports that have associated detection of coronaviruses in the CNS with acute encephalitis or encephalomyelitis (mainly in immunocompromised or immunodeficient children).^106^, ^107^, ^108^

The aetiology of the psychiatric consequences of infection with coronavirus is likely to be multifactorial and might include the direct effects of viral infection (including brain infection), cerebrovascular disease (including in the context of a procoagulant state), the degree of physiological compromise (eg, hypoxia), the immunological response, medical interventions, social isolation, the psychological impact of a novel severe and potentially fatal illness, concerns about infecting others, and stigma. The immune response in SARS-CoV-2 infection is of interest and there might be a hyperinflammatory state similar to that seen in haemophagocytic lymphohisticytosis in which there are increased concentrations of C-reactive protein, ferritin, and interleukin-6, although this state is likely to be short lived.^109^ The link between inflammation and depression is well described and might explain some of the psychiatric morbidity.^110^

Survivors of critical illness are at risk of persistent psychiatric impairment after discharge from hospital. At 1 year, the pooled prevalences of clinically relevant depressive, anxiety, and post-traumatic symptoms were 29% (23–34),^4^ 34% (25–42),^5^ and 34% (22–50),^6^ respectively. The majority of patients with severe acute respiratory distress syndrome, a key feature of severe COVID-19 illness, show impairments of memory, attention, concentration, or mental processing speed at 1 year.^111^ None of the studies included in this review completed systematic neuropsychological assessments apart from one report of severe SARS-CoV-2 cases, which described a dysexecutive syndrome in a third of survivors.^61^ Acute respiratory distress syndrome and prolonged mechanical ventilation are also associated with greater reductions in quality of life than ICU admissions for other reasons.^112^

Limitations include the use of preprint articles that have not been subject to peer review, exclusion of non-English-language articles, and the inclusion of studies with very small samples. A further limitation was that most studies were of low or moderate quality. Almost all of the studies we included in this review reported outcomes from patients admitted to hospital, which improves the comparability between coronavirus infections. However, although the frequencies of ICU admission and ventilation were similar for patients admitted to hospital with SARS-CoV infection (13% ICU admission and 7% ventilation) and SARS-CoV-2 (18% and 6%), they were considerably higher in patients with MERS (60% and 51%). Systematic assessment of psychiatric symptoms was rare, use of self-report questionnaires was common, and there was variation in the definition of illness and laboratory verification of infection between studies. The lack of baseline psychiatric assessments means that accurate estimates of incidence are impossible; therefore, we relied on point prevalence where possible. Few studies included objective biological measures, such as peripheral blood markers of genetic, inflammatory, immune, and metabolic function, cerebrospinal fluid measures, EEG, or brain imaging. Furthermore, few studies included comparison groups. The apparently high prevalence of common symptoms reported (such as depression, anxiety, and fatigue) could have been unrelated to the coronavirus infection and rather a consequence of selection bias. For the post-illness studies, there was substantial variation in follow-up time that hindered comparability. These factors might have contributed to heterogeneity, but there were too few studies to explore explanations of this variance.

Future studies should systematically assess the prevalence of psychiatric symptoms in patients with coronavirus infections, and we suggest that a prospective cohort of patients with SARS-CoV-2 should be established. Ideally, there should be measures of mental health before infection, as well as other possible confounding factors, potentially using existing cohorts. A comparison group of other patients undergoing acute medical admissions would be helpful. There would need to be standardised measures of psychiatric disorder.

It will be important to establish whether markers of severity of infection correlate with psychiatric presentations. Case-control studies of immunoreactivity to the SARS-CoV-2 virus in psychiatric populations using serological measures, once available, will give an indication of whether infection is a risk factor for psychiatric disorders.

Given that a very large number of individuals will be infected with SARS-CoV-2, the immediate impact on mental health could be considerable. An acute rise in cases of delirium will probably prolong hospital stay; there is also some preliminary evidence that delirium was associated with raised mortality in MERS.^113^ There is a risk of common mental illnesses in patients with disease that require hospital admission, which might be compounded by the effects of social isolation.^16^ Given this psychiatric morbidity and high frequency of persistent fatigue, some patients might have difficulty in returning to their previous employment, at least in the short term, although physical—as well as mental—recovery is intrinsic to such a broad functional outcome.

In conclusion, although there are many ways in which mental health might be adversely affected by a pandemic, this review suggests, first, that most people do not suffer from a psychiatric disorder following coronavirus infection, and second, that so far there is little to suggest that common neuropsychiatric complications beyond short-term delirium are a feature. Clinicians must be aware of the possibility of depression, anxiety, fatigue, post-traumatic stress disorder, and rarer neuropsychiatric syndromes in the aftermath. The quality of studies to date has been variable, and ongoing surveillance is essential.
