# Supplementary material for: Psychiatric and neuropsychiatric presentations associated with severe coronavirus infections: a systematic review and meta-analysis with comparison to the COVID-19 pandemic
Source: Lancet Psychiatry. 2020 Jul;7(7):611–27. doi: 10.1016/S2215-0366(20)30203-0 (PMC7234781; doi:10.1016/S2215-0366(20)30203-0)
Supplement: Supplementary appendix [file mmc1.pdf]

THE LANCET
Oncology

Supplementary appendix

This appendix formed part of the original submission and has been peer reviewed. We post it as supplied by the authors.

Supplement to: Rogers JP, Chesney E, Oliver D, et al. Psychiatric and neuropsychiatric presentations associated with severe coronavirus infections: a systematic review and meta-analysis with comparison to the COVID-19 pandemic. *Lancet Oncol* 2020; published online May 18. http://dx.doi.org/10.1016/S2215-0366(20)30203-0.

Appendix

Psychiatric and neuropsychiatric presentations associated with severe coronavirus infections: a systematic review and meta-analysis with comparison to the COVID-19 pandemic

Jonathan P Rogers, Edward Chesney, Dominic Oliver, Thomas Pollak, Philip McGuire, Paolo Fusar-Poli, Michael Zandi, Glyn Lewis, Anthony S David

eTable 1. PRISMA guidelines for meta-analysis and systematic reviews
eMethods 1. Full search terms
eTable 2. Scoring for Adapted Newcastle Ottawa Scale
eFigure 1 Forest plot of the mean difference of SF-36 subscales
eResults 1. Mean scores on psychiatric symptoms scales post-infection
eResults 2. Mean scores on SF-36 subscales post-infection
eResults 3. Employment
eResults 4. ICU admission and ventilation rates for SARS-CoV-1, MERS and SARS-CoV-2
eResults 5. Heterogeneity and sensitivity analyses
eTable 3. Results of quality assessment

**eTable 1:** PRISMA guidelines for meta-analysis and systematic reviews			
**Section/topic**	**#**	**Checklist item**	**Page**
**TITLE**			
Title	1	Identify the report as a systematic review, meta-analysis, or both.	1
**ABSTRACT**			
Structured summary	2	Provide a structured summary including, as applicable: background; objectives; data sources; study eligibility criteria, participants, and interventions; study appraisal and synthesis methods; results; limitations; conclusions and implications of key findings; systematic review registration number.	1
**INTRODUCTION**			
Rationale	3	Describe the rationale for the review in the context of what is already known.	2
Objectives	4	Provide an explicit statement of questions being addressed with reference to participants, interventions, comparisons, outcomes, and study design (PICOS).	2
**METHODS**			
Protocol and registration		Indicate if a review protocol exists, if and where it can be accessed (e.g., Web address), and, if available, provide	3

	5	registration information including registration number.	
Eligibility criteria	6	Specify study characteristics (e.g., PICOS, length of follow-up) and report characteristics (e.g., years considered, language, publication status) used as criteria for eligibility, giving rationale.	3
Information sources	7	Describe all information sources (e.g., databases with dates of coverage, contact with study authors to identify additional studies) in the search and date last searched.	3
Search	8	Present full electronic search strategy for at least one database, including any limits used, such that it could be repeated.	eMethods 1
Study selection	9	State the process for selecting studies (i.e., screening, eligibility, included in systematic review, and, if applicable, included in the meta-analysis).	3
Data collection process	10	Describe method of data extraction from reports (e.g., piloted forms, independently, in duplicate) and any processes for obtaining and confirming data from investigators.	3
Data items	11	List and define all variables for which data were sought (e.g., PICOS, funding sources) and any assumptions and simplifications made.	3/4
Risk of bias in individual studies	12	Describe methods used for assessing risk of bias of individual studies (including specification of whether this was done at the study or outcome level), and how this information is to be used in any data synthesis.	4
Summary measures	13	State the principal summary measures (e.g., risk ratio, difference in means).	4
Synthesis of results	14	Describe the methods of handling data and combining results of studies, if done, including measures of consistency (e.g., I^2) for each meta-analysis.	3/4
Risk of bias across studies	15	Specify any assessment of risk of bias that may affect the cumulative evidence (e.g., publication bias, selective reporting within studies).	4

Additional analyses	16	Describe methods of additional analyses (e.g., sensitivity or subgroup analyses, meta-regression), if done, indicating which were pre-specified.	4
**RESULTS**			
Study selection	17	Give numbers of studies screened, assessed for eligibility, and included in the review, with reasons for exclusions at each stage, ideally with a flow diagram.	5
Study characteristics	18	For each study, present characteristics for which data were extracted (e.g., study size, PICOS, follow-up period) and provide the citations.	6-11
Risk of bias within studies	19	Present data on risk of bias of each study and, if available, any outcome level assessment (see item 12).	15
Results of individual studies	20	For all outcomes considered (benefits or harms), present, for each study: (a) simple summary data for each intervention group (b) effect estimates and confidence intervals, ideally with a forest plot.	eFigure 1
Synthesis of results	21	Present results of each meta-analysis done, including confidence intervals and measures of consistency.	13
Risk of bias across studies	22	Present results of any assessment of risk of bias across studies (see Item 15).	eResults 1-2
Additional analysis	23	Give results of additional analyses, if done (e.g., sensitivity or subgroup analyses, meta-regression [see Item 16]).	eResults 2
**DISCUSSION**			
Summary of evidence	24	Summarize the main findings including the strength of evidence for each main outcome; consider their relevance to key groups (e.g., healthcare providers, users, and policy makers).	16
Limitations	25	Discuss limitations at study and outcome level (e.g., risk of bias), and at review-level (e.g., incomplete retrieval of identified research, reporting bias).	16/17
Conclusions	26	Provide a general interpretation of the results in the context of other evidence, and implications for future research.	17

**FUNDING**			
Funding	27	Describe sources of funding for the systematic review and other support (e.g., supply of data); role of funders for the systematic review.	17

eMethods 1. Full search terms

Medline

(((coronavir* OR alphacoronavirus OR betacoronavirus OR COVID OR COVID-19 OR "severe acute respiratory syndrome" OR SARS OR "Middle East respiratory syndrome" OR MERS OR "infectious bronchitis vir*" OR "infectious bronchitis").ti,ab OR (exp CORONAVIRIDAE/ OR exp "SEVERE ACUTE RESPIRATORY SYNDROME"/)) AND ((deliri* OR sleep OR insomnia OR somnolence OR hypersomnolence OR parasomnia OR "movement disorder" OR neuropsych* OR dement* OR cogniti* OR irritability OR hallucinat* OR delusion* OR apath* OR indifference OR agitat* OR euphori* OR elation OR elated OR disinhibit* OR aggressi* OR amnes* OR catatoni* OR personality OR psycho* OR mental OR mood OR affective OR depress* OR anxi* OR "obsessive compulsive" OR OCD OR "panic disorder" OR post-trauma* OR posttrauma* OR PTSD OR neurosis OR neurotic OR bipolar OR mania OR manic OR schizophreni* OR "intelligence quotient" OR IQ OR "mental retardation" OR "intellectual disability" OR "learning disability" OR autis* OR asperger* OR "attention deficit" OR ADHD OR hyperactivity OR hyperkinetic OR suicid* OR emotion* OR appetite OR fatigu* OR tired* OR confus*).ti,ab OR (exp DELIRIUM/ OR exp SLEEP/ OR exp WAKEFULNESS/ OR exp SLEEP/ OR exp "DISORDERS OF EXCESSIVE SOMNOLENCE"/ OR exp PARASOMNIAS/ OR exp "PSYCHOMOTOR DISORDERS"/ OR exp DEMENTIA/ OR exp "NEUROCOGNITIVE DISORDERS"/ OR exp HALLUCINATIONS/ OR exp DELUSIONS/ OR exp APATHY/ OR exp "PSYCHOMOTOR AGITATION"/ OR exp EUPHORIA/ OR exp AGGRESSION/ OR exp AMNESIA/ OR exp CATATONIA/ OR exp "PERSONALITY DISORDERS"/ OR exp "SCHIZOPHRENIA SPECTRUM AND OTHER PSYCHOTIC DISORDERS"/ OR exp "MENTAL DISORDERS"/ OR exp "MOOD DISORDERS"/ OR exp DEPRESSION/ OR exp ANXIETY/ OR exp "ANXIETY DISORDERS"/ OR exp "OBSESSIVE-COMPULSIVE DISORDER"/ OR exp "PANIC DISORDER"/ OR exp "STRESS DISORDERS, POST-TRAUMATIC"/ OR exp "BIPOLAR AND RELATED DISORDERS"/ OR exp SCHIZOPHRENIA/ OR exp "INTELLECTUAL DISABILITY"/ OR exp "AUTISM SPECTRUM DISORDER"/ OR exp "ASPERGER SYNDROME"/ OR exp "ATTENTION DEFICIT AND DISRUPTIVE BEHAVIOR DISORDERS"/ OR exp "ATTENTION DEFICIT DISORDER WITH HYPERACTIVITY"/ OR exp "MOTOR ACTIVITY"/ OR exp SUICIDE/ OR exp EMOTIONS/ OR exp APPETITE/ OR exp "FEEDING AND EATING DISORDERS"/ OR exp FATIGUE/ OR exp CONFUSION/))) [Humans]

EMBASE

(((coronavir* OR alphacoronavirus OR betacoronavirus OR COVID OR COVID-19 OR "severe acute respiratory syndrome" OR SARS OR "Middle East respiratory syndrome" OR MERS OR "infectious bronchitis vir*" OR "infectious bronchitis").ti,ab OR (exp CORONAVIRIDAE/ OR exp "CORONAVIRIDAE INFECTION"/ OR exp "SARS-RELATED CORONAVIRUS"/ OR exp "SEVERE ACUTE RESPIRATORY SYNDROME"/ OR exp "MIDDLE EAST RESPIRATORY SYNDROME"/)) AND ((deliri* OR sleep OR insomnia OR somnolence OR hypersomnolence OR parasomnia OR "movement disorder" OR neuropsych* OR dement* OR cogniti* OR irritability OR hallucinat* OR delusion* OR apath* OR indifference OR agitat* OR euphori* OR elation OR elated OR disinhibit* OR aggressi* OR amnes* OR catatoni* OR personality OR psycho* OR mental OR mood OR affective OR depress* OR anxi* OR "obsessive compulsive" OR OCD OR "panic disorder" OR post-trauma* OR posttrauma* OR PTSD OR neurosis OR neurotic OR bipolar OR mania OR manic OR schizophreni* OR "intelligence quotient" OR IQ OR "mental retardation" OR "intellectual disability" OR "learning disability" OR autis* OR asperger* OR "attention deficit" OR ADHD OR hyperactivity OR hyperkinetic OR suicid* OR emotion* OR appetite OR fatigu* OR tired* OR confus*).ti,ab OR (exp DELIRIUM/ OR exp "SLEEP DISORDER"/ OR exp INSOMNIA/ OR exp SOMNOLENCE/ OR exp HYPERSOMNIA/ OR exp PARASOMNIA/ OR exp "MOTOR DYSFUNCTION"/ OR exp DEMENTIA/ OR exp "COGNITIVE DEFECT"/ OR exp IRRITABILITY/ OR exp HALLUCINATION/ OR exp DELUSION/ OR exp APATHY/ OR exp AGITATION/ OR exp EUPHORIA/ OR exp AGGRESSION/ OR exp AMNESIA/ OR exp CATATONIA/ OR exp "PERSONALITY DISORDER"/ OR exp PSYCHOSIS/ OR exp "MENTAL DISEASE"/ OR exp MOOD/ OR exp "MOOD DISORDER"/ OR exp DEPRESSION/ OR exp

ANXIETY/ OR exp "ANXIETY DISORDER"/ OR exp "OBSESSIVE COMPULSIVE DISORDER"/ OR exp PANIC/ OR exp "POSTTRAUMATIC STRESS DISORDER"/ OR exp NEUROSIS/ OR exp "BIPOLAR DISORDER"/ OR exp MANIA/ OR exp PSYCHOSIS/ OR exp "SCHIZOPHRENIA SPECTRUM DISORDER"/ OR exp SCHIZOPHRENIA/ OR exp "INTELLIGENCE QUOTIENT"/ OR exp "MENTAL DISEASE"/ OR exp "INTELLECTUAL IMPAIRMENT"/ OR exp "DISORDERS OF HIGHER CEREBRAL FUNCTION"/ OR exp "LEARNING DISORDER"/ OR exp AUTISM/ OR exp "ATTENTION DEFICIT DISORDER"/ OR exp HYPERACTIVITY/ OR exp "PSYCHOMOTOR DISORDER"/ OR exp HYPERKINESIA/ OR exp SUICIDE/ OR exp "SUICIDAL BEHAVIOR"/ OR exp "SUICIDE ATTEMPT"/ OR exp EMOTION/ OR exp APPETITE/ OR exp "APPETITE DISORDER"/ OR exp FATIGUE/ OR exp CONFUSION/))) [Humans]

PsycINFO
((coronavir* OR alphacoronavirus OR betacoronavirus OR COVID OR COVID-19 OR "severe acute respiratory syndrome" OR SARS OR "Middle East respiratory syndrome" OR MERS OR "infectious bronchitis vir*" OR "infectious bronchitis").ti,ab AND ((deliri* OR sleep OR insomnia OR somnolence OR hypersomnolence OR parasomnia OR "movement disorder" OR neuropsych* OR dement* OR cogniti* OR irritability OR hallucinat* OR delusion* OR apath* OR indifference OR agitat* OR euphori* OR elation OR elated OR disinhibit* OR aggressi* OR amnes* OR catatoni* OR personality OR psycho* OR mental OR mood OR affective OR depress* OR anxi* OR "obsessive compulsive" OR OCD OR "panic disorder" OR post-trauma* OR posttrauma* OR PTSD OR neurosis OR neurotic OR bipolar OR mania OR manic OR schizophreni* OR "intelligence quotient" OR IQ OR "mental retardation" OR "intellectual disability" OR "learning disability" OR autis* OR asperger* OR "attention deficit" OR ADHD OR hyperactivity OR hyperkinetic OR suicid* OR emotion* OR appetite OR fatigu* OR tired* OR confus*).ti,ab OR (exp DELIRIUM/ OR exp "NEUROCOGNITIVE DISORDERS"/ OR exp "SLEEP WAKE DISORDERS"/ OR exp INSOMNIA/ OR exp HYPERSOMNIA/ OR exp "MOVEMENT DISORDERS"/ OR exp DEMENTIA/ OR exp "COGNITIVE ABILITY"/ OR exp "COGNITIVE IMPAIRMENT"/ OR exp "NEUROCOGNITIVE DISORDERS"/ OR exp IRRITABILITY/ OR exp HALLUCINATIONS/ OR exp DELUSIONS/ OR exp APATHY/ OR exp AGITATION/ OR exp EUPHORIA/ OR exp "BEHAVIORAL DISINHIBITION"/ OR exp AMNESIA/ OR exp CATATONIA/ OR exp PERSONALITY/ OR exp "PERSONALITY DISORDERS"/ OR exp PSYCHOSIS/ OR exp "MENTAL DISORDERS"/ OR exp EMOTIONS/ OR exp "AFFECTIVE DISORDERS"/ OR exp "DEPRESSION (EMOTION)"/ OR exp ANXIETY/ OR exp "ANXIETY DISORDERS"/ OR exp "OBSESSIVE COMPULSIVE DISORDER"/ OR exp "PANIC DISORDER"/ OR exp "POST-TRAUMATIC STRESS"/ OR exp NEUROSIS/ OR exp "BIPOLAR DISORDER"/ OR exp MANIA/ OR exp SCHIZOPHRENIA/ OR exp "INTELLIGENCE QUOTIENT"/ OR exp "NEURODEVELOPMENTAL DISORDERS"/ OR exp "INTELLECTUAL DEVELOPMENT DISORDER"/ OR exp "AUTISM SPECTRUM DISORDERS"/ OR exp "ATTENTION DEFICIT DISORDER"/ OR exp HYPERKINESIS/ OR exp SUICIDE/ OR exp EMOTIONS/ OR exp APPETITE/ OR exp "EATING DISORDERS"/ OR exp FATIGUE/ OR exp "MENTAL CONFUSION"/))) [Population Human]

CINAHL
((coronavir* OR alphacoronavirus OR betacoronavirus OR COVID OR COVID-19 OR "severe acute respiratory syndrome" OR SARS OR "Middle East respiratory syndrome" OR MERS OR "infectious bronchitis vir*" OR "infectious bronchitis").ti,ab OR (exp CORONAVIRUS/ OR exp CORONAVIRIDAE/ OR exp "MIDDLE EAST RESPIRATORY SYNDROME CORONAVIRUS"/ OR exp "SARS VIRUS"/)) AND ((deliri* OR sleep OR insomnia OR somnolence OR hypersomnolence OR parasomnia OR "movement disorder" OR neuropsych* OR dement* OR cogniti* OR irritability OR hallucinat* OR delusion* OR apath* OR indifference OR agitat* OR euphori* OR elation OR elated OR disinhibit* OR aggressi* OR amnes* OR catatoni* OR personality OR psycho* OR mental OR mood OR affective OR depress* OR anxi* OR "obsessive compulsive" OR OCD OR "panic disorder" OR post-trauma* OR posttrauma* OR PTSD OR neurosis OR neurotic OR bipolar OR mania OR manic OR schizophreni* OR "intelligence quotient" OR IQ

OR "mental retardation" OR "intellectual disability" OR "learning disability" OR autis* OR asperger* OR "attention deficit" OR ADHD OR hyperactivity OR hyperkinetic OR suicid* OR emotion* OR appetite OR fatigu* OR tired* OR confus*).ti,ab OR (exp DELIRIUM/ OR exp SLEEP/ OR exp "SLEEP DISORDERS"/ OR exp INSOMNIA/ OR exp "DISORDERS OF EXCESSIVE SOMNOLENCE"/ OR exp PARASOMNIAS/ OR exp "MOVEMENT DISORDERS"/ OR exp DEMENTIA/ OR exp "COGNITION DISORDERS"/ OR exp "DELIRIUM, DEMENTIA, AMNESTIC, COGNITIVE DISORDERS"/ OR exp HALLUCINATIONS/ OR exp DELUSIONS/ OR exp APATHY/ OR exp AGITATION/ OR exp AGGRESSION/ OR exp AMNESIA/ OR exp "MEMORY DISORDERS"/ OR exp CATATONIA/ OR exp PERSONALITY/ OR exp "PERSONALITY DISORDERS"/ OR exp "PERSONALITY DISORDERS"/ OR exp "PERSONALITY DISORDERS"/ OR exp "PSYCHOTIC DISORDERS"/ OR exp "MENTAL DISORDERS"/ OR exp "AFFECTIVE SYMPTOMS"/ OR exp "AFFECTIVE DISORDERS"/ OR exp DEPRESSION/ OR exp ANXIETY/ OR exp "ANXIETY DISORDERS"/ OR exp "NEUROTIC DISORDERS"/ OR exp "OBSESSIVE-COMPULSIVE DISORDER"/ OR exp "PANIC DISORDER"/ OR exp "STRESS DISORDERS, POST-TRAUMATIC"/ OR exp "BIPOLAR DISORDER"/ OR exp SCHIZOPHRENIA/ OR exp "INTELLECTUAL DISABILITY"/ OR exp "AUTISTIC DISORDER"/ OR exp "CHILD DEVELOPMENT DISORDERS, PERVASIVE"/ OR exp "ASPERGER SYNDROME"/ OR exp "ATTENTION DEFICIT HYPERACTIVITY DISORDER"/ OR exp HYPERKINESIS/ OR exp "MOTOR ACTIVITY"/ OR exp SUICIDE/ OR exp EMOTIONS/ OR exp APPETITE/ OR exp "EATING DISORDERS"/ OR exp FATIGUE/ OR exp CONFUSION/))

PsyArXiv
Terms: coronavirus OR covid OR "covid-19"
From inception to date (5 hits; all since March 2020)
Search completed on 17/3/20

bioRxiv and medRxiv
Terms: for full text or abstract or title "coronavirus, covid-19" (match whole any) and posted between "01 Jan, 2020 and 17 Mar, 2020"
627 hits at 10.30pm on 17/3/2020

eTable 2. Scoring for Adapted Newcastle Ottawa Scale

**Selection 1**	Representativeness of cohort	1) Typical hospitalised coronavirus<br>0) <70% recruitment or >30% lost<br>0) community cases<br>0) Intensive care only<br>0) Healthcare workers only<br>0) ITU
**Selection 2**	Standard Criteria, and does not include suspected cases	1) Probable/Confirmed only according to WHO/CDC/relevant criteria<br>0) Suspected
**Selection 3**	Ascertainment of exposure	1) Lab verification<br>0) No lab test
**Selection 4**	Outcome of interest was not present at start of study	1) Psychiatric assessment pre-coronavirus<br>0) No psychiatric assessment
**Comparability**	Control group?	2) Well matched healthy controls<br>1) Age-matched normative data; poorly matched healthy controls<br>0) drawn from a different source<br>0) no description of the derivation of the non-exposed cohort<br>0) normative data
**Outcome 1**	Systematic assessment	1) All participants assessed<br>0) Targeted/unstructured

**Outcome 2**	Quality of assessment	1) Clinician diagnosis using ICD-10/DSMIV<br>1) Systematic symptoms assessment<br>1) Standardised rating scales<br>1) High-quality qualitative methods<br>0) Scales only used to obtain diagnosis i.e. not also in a normal way<br>0) Unstructured reporting of diagnosis, symptoms or severity
**Outcome 3**	Follow-up	1) Same time-point for all, adequate follow-up time adequate<br>0) Follow-up <3 months<br>0) Not all patients discharged/dead

eFigure 1 Forest plot of the mean difference of SF-36 subscales **A** social functioning, **B** role limitation due to emotional problems, and **C** mental health

A

Study or Subgroup	Coronavirus Infection Mean	SD	Total	Control Mean	SD	Total	Weight	Mean Difference IV, Random, 95% CI
Hui 2005 (Age 18–40) (7)	72.6	23.3	35	90.2	16.2	415	32.0%	−17.60 [−25.47, −9.73]
Hui 2005 (Age 41–64) (7)	55.3	26.5	62	92.4	16.5	232	33.3%	−37.10 [−44.03, −30.17]
Mak 2009 (924)	67.1	27.8	90	91.2	16.6	803	34.7%	−24.10 [−29.96, −18.24]
**Total (95% CI)**			**187**			**1450**	**100.0%**	**−26.35 [−37.01, −15.69]**

Heterogeneity: $Tau^2 = 76.34$; $Chi^2 = 14.54$, df = 2 (P = 0.0007); $I^2 = 86\%$
Test for overall effect: Z = 4.84 (P < 0.00001)

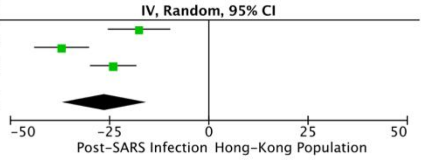

B

Study or Subgroup	Coronavirus Infection Mean	SD	Total	Control Mean	SD	Total	Weight	Mean Difference IV, Random, 95% CI
Hui 2005 (Age 18–40) (7)	70.1	40.9	35	67.6	39.4	415	31.3%	2.50 [−11.57, 16.57]
Hui 2005 (Age 41–64) (7)	47.7	46.1	62	75	37	232	33.1%	−27.30 [−39.72, −14.88]
Mak 2009 (924)	51.7	46.4	90	71.7	38.4	803	35.7%	−20.00 [−29.95, −10.05]
**Total (95% CI)**			**187**			**1450**	**100.0%**	**−15.38 [−31.24, 0.48]**

Heterogeneity: $Tau^2 = 157.89$; $Chi^2 = 10.37$, df = 2 (P = 0.006); $I^2 = 81\%$
Test for overall effect: Z = 1.90 (P = 0.06)

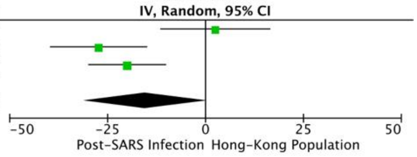

C

Study or Subgroup	Coronavirus Infection Mean	SD	Total	Control Mean	SD	Total	Weight	Mean Difference IV, Random, 95% CI
Hui 2005 (Age 18–40) (7)	65.1	18.4	35	71.8	15.6	415	24.3%	−6.70 [−12.98, −0.42]
Hui 2005 (Age 41–64) (7)	60.3	19.2	62	73.1	17.6	232	33.2%	−12.80 [−18.09, −7.51]
Mak 2009 (924)	61.6	21.6	90	72.8	16.6	803	42.5%	−11.20 [−15.81, −6.59]
**Total (95% CI)**			**187**			**1450**	**100.0%**	**−10.64 [−13.85, −7.43]**

Heterogeneity: $Tau^2 = 0.78$; $Chi^2 = 2.21$, df = 2 (P = 0.33); $I^2 = 10\%$
Test for overall effect: Z = 6.50 (P < 0.00001)

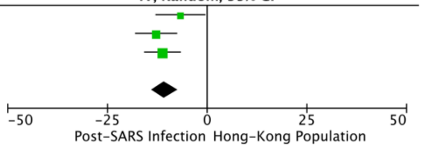

eResults 1. Mean scores on psychiatric symptoms scales post-infection **A** anxiety, **B** depression, **C** PTSD, **D** PTSD intrusion, **E** PTSD avoidance, and **F** PTSD hyperarousal

Anxiety symptoms

Study	Virus	Scale	Follow-up (months)	n	Mean	SD
Lam 2009	SARS-CoV-1	HADS (anxiety)	41.3 (range 31-51)	170	7.6	4.4
Kwek 2006	SARS-CoV-1	HADS (anxiety)	3	63	6.2	4.5
Wu 2005	SARS-CoV-1	HADS (anxiety)	3	131	5.19	4.48

Depression symptoms

Study	Virus	Scale	Follow-up (months)	n	Mean	SD
Lam 2009	SARS-CoV-1	HADS (depression)	41.3 (range 31-51)	170	7.4	4.5
Kwek 2006	SARS-CoV-1	HADS (depression)	3	63	4.9	4.1
Wu 2005	SARS-CoV-1	HADS (depression)	3	131	5.17	4.33

PTSD symptoms

Study	Virus	Scale	Follow-up (months)	n	Mean	SD
Lee 2019	MERS	IES-R (total)	18	52	19.29	21.03
Kwek 2006	SARS-CoV-1	IES (total)	3	63	21.8	16.3

PTSD intrusion symptoms

Study	Virus	Scale	Follow-up (months)	n	Mean	SD
Lam 2009	SARS-CoV-1	IES-R (intrusion)	41.3 (range 31-51)	170	13.28	8
Lee 2007	SARS-CoV-1	IES-R (intrusion)	12	96	11.2	7.2
Wu 2005	SARS-CoV-1	IES-R (intrusion)	3	131	7.28	5.84

PTSD avoidance symptoms

Study	Virus	Scale	Follow-up (months)	n	Mean	SD
Lam 2009	SARS-CoV-1	IES-R (avoidance)	41.3 (range 31-51)	170	10.16	7.76
Lee 2007	SARS-CoV-1	IES-R (avoidance)	12	96	8.8	6.4
Wu 2005	SARS-CoV-1	IES-R (avoidance)	3	131	6.96	5.76

PTSD hyperarousal symptoms

Study	Virus	Scale	Follow-up (months)	n	Mean	SD
Lam 2009	SARS-CoV-1	IES-R (hyperarousal)	41.3 (range 31-51)	170	10.56	6.24
Lee 2007	SARS-CoV-1	IES-R (hyperarousal)	12	96	7.8	5.4
Wu 2005	SARS-CoV-1	IES-R (hyperarousal)	3	131	5.1	4.44

eResults 2. Mean scores on SF-36 subscales post infection **A** social functioning, **B** role limitation due to emotional problems, and **C** mental health

**Quality of Life - Social Functioning**						
**Study**	**Virus**	**Scale**	**Follow-up (months)**	**n**	**Mean**	**SD**
Hui 2005	SARS-CoV-1	SF-36: Social Functioning	12	19	72.4	24.1
Hui 2005	SARS-CoV-1	SF-36: Social Functioning	12	12	70.8	27.9
Hui 2005	SARS-CoV-1	SF-36: Social Functioning	12	50	72.8	23.1
Hui 2005	SARS-CoV-1	SF-36: Social Functioning	12	16	51.6	25
Mak 2009	SARS-CoV-1	SF-36: Social Functioning	18	143	68.25	27
Lam 2006	SARS-CoV-1	SF-36: Social Functioning	2	110	55.23	29.44
Hong 2009	SARS-CoV-1	SF-36: Social Functioning	10	22	50.66	31.31
Hong 2009	SARS-CoV-1	SF-36: Social Functioning	10	35	80.15	25.58
Mak 2009	SARS-CoV-1	SF-36: Social Functioning	30	90	67.07	27.81
Kwek 2006	SARS-CoV-1	SF-36: Social Functioning	3	6	79.6	28.4
Batawi 2019	MERS	SF-36: Social Functioning	12	78	84.45	24.36

**Quality of Life - Role Limitation Due to Emotional Problems**						
**Study**	**Virus**	**Scale**	**Follow-up (months)**	**n**	**Mean**	**SD**
Hui 2005	SARS-CoV-1	SF-36: Role Limitation Due to Emotional Problems	12	19	71.9	42
Hui 2005	SARS-CoV-1	SF-36: Role Limitation Due to Emotional Problems	12	12	55.6	43.4
Hui 2005	SARS-CoV-1	SF-36: Role Limitation Due to Emotional Problems	12	50	68	40.9
Hui 2005	SARS-CoV-1	SF-36: Role Limitation Due to Emotional Problems	12	16	45.8	46.9
Mak 2009	SARS-CoV-1	SF-36: Role Limitation Due to Emotional Problems	18	143	3.59	1.07
Lam 2006	SARS-CoV-1	SF-36: Role Limitation Due to Emotional Problems	2	112	37.5	40.3
Hong 2009	SARS-CoV-1	SF-36: Role Limitation Due to Emotional Problems	10	22	33.33	41.57
Hong 2009	SARS-CoV-1	SF-36: Role Limitation Due to Emotional Problems	10	35	89.22	26.87
Mak 2009	SARS-CoV-1	SF-36: Role Limitation Due to Emotional Problems	30	90	51.7	46.35
Kwek 2006	SARS-CoV-1	SF-36: Role Limitation Due to Emotional Problems	3	6	77.8	40.4
Batawi 2019	MERS	SF-36: Role Limitation Due to Emotional Problems	12	78	75.12	36.39

**Quality of Life - Mental Health**						
**Study**	**Virus**	**Scale**	**Follow-up (months)**	**n**	**Mean**	**SD**
Hui 2005	SARS-CoV-1	SF-36: Mental Health	12	19	63.4	18.3
Hui 2005	SARS-CoV-1	SF-36: Mental Health	12	12	66.3	20.6
Hui 2005	SARS-CoV-1	SF-36: Mental Health	12	50	67.2	18.8
Hui 2005	SARS-CoV-1	SF-36: Mental Health	12	16	58.8	18.8
Mak 2009	SARS-CoV-1	SF-36: Mental Health	18	143	3.45	0.82
Lam 2006	SARS-CoV-1	SF-36: Mental Health	2	112	64.68	21.36
Hong 2009	SARS-CoV-1	SF-36: Mental Health	10	22	54.11	19.43
Hong 2009	SARS-CoV-1	SF-36: Mental Health	10	35	80	15.94

Mak 2009	SARS-CoV-1	SF-36: Mental Health	30	90	61.62	21.57
Kwek 2006	SARS-CoV-1	SF-36: Mental Health	3	6	88	15.8
Batawi 2019	MERS	SF-36: Mental Health	12	78	79.64	22.34

eResults 3. Employment

**Employment**				
**Study**	**Virus**	**Follow-up (months)**	**n**	**Employed n (%)**
Mak 2009	SARS-Cov-1	18	143	94 (66%)
Guo 2019	SARS-Cov-1	144	67	62 (93%)
Lee 2019	MERS	12	52	43 (83%
Lam 2009	SARS-Cov-1	41.3	170	116 (68%)
Tansey 2007	SARS-Cov-1	1	107	79 (74%)
Ngai 2010	SARS-Cov-1	24	41	32 (78%)

eResults 4. ICU admission and ventilation rates for SARS-CoV-1, MERS and SARS-CoV-2

SARS-CoV-1 ICU admission rate: 12.8% (k = 5, n=37, 95% CI 6.9% to 22.5%)
SARS-CoV-1 ventilation rate: 6.5% (k=5, n=15, 95% CI 3.4% to 11.9%)

MERS ICU admission rate: 59.8% (k = 2, n=102, 95% CI 38.9% to 77.6%)
MERS ventilation rate: 50.8% (k = 3, n=113, 95% CI 30.5% to 70.1%)

SARS-CoV-2 ICU admission rate: 18.3% (k = 4, n=93, 95% CI 13.3% to 24.6%)
SARS-CoV-2 ventilation rate: 5.8% (k = 4, n=22, 95% CI 1.6% to 19.0%)

eResults 5. Heterogeneity and sensitivity analyses

Anxiety: Heterogeneity was low ($I^2 = 0$) with no studies significantly changing the summary effect if excluded in sensitivity analyses ($p>0.05$).

Depression: Heterogeneity was low ($I^2 = 0$) with no studies significantly changing the summary effect if excluded in sensitivity analyses ($p>0.05$).

PTSD: Heterogeneity was high ($I^2 = 80\%$) with all studies significantly changing the summary effect if excluded in sensitivity analyses ($p<0.001$). Despite heterogeneity being high, there were not sufficient studies to conduct meta-regression to explain this variance.

eTable 3. Results of quality assessment

	First author	Year	Title	Quality category: Low 0-3 Medium 4-6 High 7-9
**1**	Hui	2005	The 1-year impact of severe acute respiratory syndrome on pulmonary function, exercise capacity, and quality of life in a cohort of survivors.	medium
**2**	Mak	2009	Social support and psychological adjustment to SARS: the mediating role of self-care self-efficacy.	low
**3**	Avendano	2003	Clinical course and management of SARS in health care workers in Toronto: a case series.	low
**4**	Koller	2006	When family-centered care is challenged by infectious disease: pediatric health care delivery during the SARS outbreaks.	medium
**5**	Wing	2012	Mental health impact of severe acute respiratory syndrome: a prospective study.	low
**6**	Han	2003	A follow-up study of 69 discharged SARS patients.	low
**7**	Tso	2004	Persistence of physical symptoms in and abnormal laboratory findings for survivors of severe acute respiratory syndrome.	low
**8**	Lo	2005	Autonomic dysfunction in recovered severe acute respiratory syndrome patients.	high
**9**	Lam	2006	The validity and reliability of the functional impairment checklist (FIC) in the evaluation of functional consequences of severe acute respiratory distress syndrome (SARS).	low
**10**	Almutairi	2018	"It feels like I'm the dirtiest person in the world.": Exploring the experiences of healthcare providers who survived MERS-CoV in Saudi Arabia.	medium
**11**	Chen	2020	Epidemiological and clinical characteristics of 99 cases of 2019 novel coronavirus pneumonia in Wuhan, China: a descriptive study.	low
**12**	Guo	2019	Long-term outcomes in patients with severe acute respiratory syndrome treated with oseltamivir: A 12-year longitudinal study	medium
**13**	Lee	2019	Depression as a mediator of chronic fatigue and post-traumatic stress symptoms in middle east respiratory syndrome survivors	medium
**14**	Hong	2018	Predictors of mortality in Middle East respiratory syndrome (MERS)	medium
**15**	Kim	2018	Psychiatric findings in suspected and confirmed middle east respiratory syndrome patients quarantined in hospital: A retrospective chart analysis	medium
**16**	Alhumaid	2018	MERS-coV transmitted from animal-to-human vs MERS-CoV transmitted from human-to-human: Comparison of virulence and therapeutic outcomes in a Saudi Hospital	medium
**17**	Lee	2017	The clinical and virological features of the first imported case causing MERS-CoV outbreak in South Korea, 2015	medium
**18**	Mak	2010	Risk factors for chronic post-traumatic stress disorder (PTSD) in SARS survivors.	medium
**19**	Yoon	2016	System effectiveness of detection, brief intervention and refer to treatment for the people with post-traumatic emotional distress by MERS: A case report of community-based proactive intervention in South Korea	low
**20**	Siu	2016	Coping with future epidemics: Tai chi practice as an overcoming strategy used by survivors of severe acute respiratory syndrome (SARS) in post-SARS Hong Kong	low
**21**	Noorwali	2015	Descriptive epidemiology and characteristics of confirmed cases of Middle East respiratory syndrome coronavirus infection in the Makkah Region of Saudi Arabia, March to June 2014	medium
**22**	Saad	2014	Clinical aspects and outcomes of 70 patients with Middle East respiratory syndrome coronavirus infection: A single-center experience in Saudi Arabia	low
**23**	Guery	2013	Clinical features and viral diagnosis of two cases of infection with Middle East Respiratory Syndrome coronavirus: A report of nosocomial transmission	low
**24**	Moldofsky	2011	Chronic widespread musculoskeletal pain, fatigue, depression and disordered sleep in chronic post-SARS syndrome; a case-controlled study	low
**25**	Lam	2009	Mental morbidities and chronic fatigue in severe acute respiratory syndrome survivors long-term follow-up	low
**26**	Hong	2009	Posttraumatic stress disorder in convalescent severe acute respiratory syndrome patients: a 4-year follow-up study	medium
**27**	Mak	2009	Long-term psychiatric morbidities among SARS survivors	high
**28**	Siu	2008	The sars-associated stigma of SARS victims in the post-sars Era of Hong Kong	low
**29**	Bonanno	2008	Psychological Resilience and Dysfunction Among Hospitalized Survivors of the SARS Epidemic in Hong Kong: A Latent Class Approach	medium

**30**	Lee	2007	Stress and psychological distress among SARS survivors 1 year after the outbreak	low
**31**	Tansey	2007	One-year outcomes and health care utilization in survivors of severe acute respiratory syndrome	medium
**32**	Mok	2005	An exploratory study of nurses suffering from severe acute respiratory syndrome (SARS)	low
**33**	Wu	2005	Posttraumatic stress, anxiety, and depression in survivors of severe acute respiratory syndrome (SARS)	low
**34**	MacKay	2005	Adverse drug reactions associated with the use of ribavirin in the treatment of severe acute respiratory syndrome (SARS)	low
**35**	Kwek	2006	Quality of life and psychological status in survivors of severe acute respiratory syndrome at 3 months postdischarge	medium
**36**	Lau	2005	Tachycardia amongst subjects recovering from severe acute respiratory syndrome (SARS)	low
**37**	Leow	2005	Hypocortisolism in survivors of severe acute respiratory syndrome (SARS)	low
**38**	Lee	2005	The experience of SARS-related stigma at Amoy Gardens	low
**39**	Wu	2005	Posttraumatic stress after SARS	low
**40**	Sheng	2005	The effects of disease severity, use of corticosteroids and social factors on neuropsychiatric complaints in severe acute respiratory syndrome (SARS) patients at acute and convalescent phases	low
**41**	Leung	2004	Severe acute respiratory syndrome among children	low
**42**	Tiwari	2003	Severe acute respiratory syndrome (SARS) in Hong Kong: Patients' experiences	low
**43**	Cheng	2004	Adjustment outcomes in Chinese patients following one-month recovery from severe acute respiratory syndrome in Hong Kong	medium
**44**	Cheng	2004	Psychological distress and negative appraisals in survivors of severe acute respiratory syndrome (SARS)	medium
**45**	Li	2004	Long-term sequelae of SARS in children	low
**46**	Lee	2004	Factors associated with psychosis among patients with severe acute respiratory syndrome: A case-control study	low
**47**	Cheng	2004	Psychiatric complications in patients with severe acute respiratory syndrome (SARS) during the acute treatment phase: A series of 10 cases	medium
**48**	Lau	2004	Outcome of coronavirus-associated severe acute respiratory syndrome using a standard treatment protocol	medium
**49**	Chua	2004	Stress and psychological impact on SARS patients during the outbreak	medium
**50**	Loutfy	2003	Interferon Alfacon-1 Plus Corticosteroids in Severe Acute Respiratory Syndrome: A Preliminary Study	medium
**51**	Maunder	2003	The immediate psychological and occupational impact of the 2003 SARS outbreak in a teaching hospital	low
**52**	Cheng,	2006	Adjustment to severe acute respiratory syndrome (SARS): Roles of appraisal and post-traumatic growth	low
**53**	Batawi,	2019	Quality of life reported by survivors after hospitalization for Middle East Respiratory Syndrome (MERS)	medium
**54**	Jeong,	2016	Mental health status of people isolated due to Middle East Respiratory Syndrome.	medium
**55**	Arabi,	2015	Severe neurologic syndrome associated with Middle East respiratory syndrome corona virus (MERS-CoV).	low
**56**	Schneider	2004	SARS in pregnancy: this case study explores the first documented infection in the U.S.	low
**57**	JENNY	2010	The long-term impact of severe acute respiratory syndrome on pulmonary function, exercise capacity and health status	medium
**58**	Hui,	2005	Impact of severe acute respiratory syndrome (SARS) on pulmonary function, functional capacity and quality of life in a cohort of survivors	high
**59**	Lau,	2005	The impact of severe acute respiratory syndrome on the physical profile and quality of life	medium
**60**	Li,	2006	Long-term outcome of acute respiratory distress syndrome caused by severe acute respiratory syndrome (SARS): An observational study	medium
**61**	Chen	2020	Clinical and immunologic features in severe and moderate forms of Coronavirus Disease 2019	low
**62**	Zhang	2020	Clinical characteristics of 82 death cases with COVID-19	medium
**63**	Qi	2020	Epidemiological and clinical features of 2019-nCoV acute respiratory disease cases in Chongqing municipality, China: a retrospective, descriptive, multiple-center study	medium
**64**	Ying	2020	Clinical characteristics of 36 non-survivors with COVID-19 in Wuhan, China	low
**65**	Ling	2020	Neurological Manifestations of Hospitalized Patients with COVID-19 in Wuhan, China: a retrospective case series study	medium

**66**	Lau	2021	A randomised controlled trial of the effectiveness of an exercise training program in patients recovering from severe acute respiratory syndrome	medium
**67**	Leung	2020	A territory-wide study of early COVID-19 outbreak in Hong Kong community: A clinical, epidemiological and phylogenomic investigation	medium
**68**	Fu	2020	Virologic and clinical characteristics for prognosis of severe COVID-19: a retrospective observational study in Wuhan, China	low
**69**	Kong	2020	Prevalence and Factors Associated with Depression and Anxiety of Hospitalized Patients with COVID-19	medium
**70**	Yang	2020	Analysis of psychological state and clinical psychological intervention model of patients with COVID-19	high
**71**	Moriguchi	2020	A first Case of Meningitis/Encephalitis associated with SARS-Coronavirus-2	Medium
**72**	Helms	2020	Neurologic Features in Severe SARS-CoV-2 Infection	Medium
